# Polylactic co-glycolic acid (PLGA)-based nanoparticles encapsulating *Persea americana* seed extract: A potential therapeutic strategy against oxidative stress and diabetes in rat model

**DOI:** 10.1371/journal.pone.0359514

**Published:** 2026-10-22

**Authors:** Sidra Altaf, Rafia Gulnaz, Asmara, Ahmed Othman Alsabih, Rao Zahid Abbas, Mohamed Y. Zaky, Mostafa A. Abdel-Maksoud

**Affiliations:** 1 Department of Pharmacy, University of Agriculture Faisalabad, Punjab, Pakistan; 2 Institute of Physiology and Pharmacology, University of Agriculture, Faisalabad, Punjab, Pakistan; 3 Department of Physiology, College of Medicine, King Saud University, Riyadh, Saudi Arabia; 4 Department od Parasitology, University of Agriculture Faisalabad, Punjab, Pakistan; 5 UPMC Hillman Cancer Center, Division of Hematology and Oncology, Department of Medicine, University of Pitsburgh, Pennsylvania, United States of America; 6 Research Chair of Biomedical applications of nanomaterials, Biochemistry department- College of science, King Saud University, Riyadh, Saudi Arabia; Helwan University, EGYPT

## Abstract

Diabetes mellitus is a chronic metabolic disorder characterized by persistent hyperglycemia arising from insulin resistance or impaired insulin secretion. Conventional and commercially available effective antidiabetic drugs are often associated with adverse effects and reduced efficacy over prolonged use, highlighting the need for safer and plant-based alternatives. *Persea americana* seed extract (PSE) has antidiabetic potential but suffers from non-specific distribution and poor bioavailability, thus limiting its clinical application. This study developed and evaluated the poly(lactic-co-glycolic acid) (PLGA) nanoparticles encapsulating *Persea americana* seed extract (PSENP) to enhance therapeutic efficacy and delivery. Nanoparticles were characterized for zeta size, zeta potential, transmission electron microscopy (TEM), stability and their phytochemical composition, with HPLC confirming the presence of bioactive compounds including quercetin, syringic acid, and gallic acid. *In-vivo*, forty wistar rats were allocated into five groups: normal control, diabetic control, metformin-treated, PSE-treated, and PSENP-treated. Biochemical parameters were evaluated including fasting blood glucose, insulin (serum level), lipid profile, oxidative stress biomarkers (superoxide dismutase, catalase), and inflammatory cytokines (IL-6, TNF-α). Histopathological examination of pancreatic tissue and safety evaluations for kidney and liver were performed. Moreover, In-silico study demonstrated the strong binding affinity between quercetin and α-glucosidase, thus supporting the glucose-lowering role of quercetin. The PSENP administration resulted in statistically significant glycemic control, enhanced antioxidant activity of enzymes, improved lipid profile, reduced pro-inflammatory cytokines, and preservation of architecture of pancreas, with no renal and hepatic toxicity. The findings of the study indicated that PLGA-based nanoformulation of *P. americana* seed extract provides a promising therapeutic strategy for diabetes management and its oxidative and inflammatory complications.

## Introduction

Diabetes mellitus (DM) is a multifactorial and progressive metabolic disorder characterized by chronic hyperglycemia resulting from defects in insulin secretion, insulin action, or both [1]. It remains a major global health challenge and is associated with severe complications including nephropathy, neuropathy, retinopathy, and cardiovascular disorders, significantly contributing to morbidity and mortality worldwide [2,3]. Type 1 diabetes is primarily caused by autoimmune-mediated destruction of pancreatic β-cells [4,5]. Among experimental diabetic models, alloxan-induced diabetes is widely used to mimic Type 1-like diabetic conditions due to its selective cytotoxic effect on pancreatic β-cells through reactive oxygen species generation and oxidative stress-mediated damage.

Oxidative stress plays a central role in the pathogenesis and progression of diabetes and its associated complications. Excessive production of reactive oxygen species can impair pancreatic β-cell integrity, reduce insulin secretion, and exacerbate inflammatory pathways. Although several conventional antidiabetic agents, including metformin, sulfonylureas, and insulin analogs, are currently available, their prolonged use is frequently associated with adverse effects such as hypoglycemia, gastrointestinal disturbances, and reduced patient compliance [6]. Consequently, increasing attention has been directed toward natural products with antioxidant and antihyperglycemic potential as alternative therapeutic strategies [7].

Medicinal plants contain diverse bioactive compounds such as flavonoids, phenolic acids, tannins, and alkaloids that may modulate glucose metabolism, reduce oxidative stress, and improve pancreatic function [8–11]. *Persea americana* (avocado) seed, commonly considered an agricultural by-product, is a rich source of phytochemicals possessing antioxidant, anti-inflammatory, and antidiabetic activities [12]. Previous studies have demonstrated the therapeutic potential of avocado seed extracts in reducing hyperglycemia and oxidative stress [13,14]; however, their clinical applicability remains limited due to poor aqueous solubility, instability, and low bioavailability under physiological conditions [15].

To overcome these limitations, nanotechnology-based drug delivery systems have been employed [16]. The biodegradable polymers such as PLGA have emerged as promising approaches for enhancing the therapeutic performance of plant-derived bioactives [17]. PLGA nanoparticles (PLGANP) improve the stability of encapsulated compounds, protect phytochemicals from premature degradation, enhance gastrointestinal absorption, and provide controlled and sustained release profiles [18]. Encapsulation of herbal extracts within PLGA nanoparticles may therefore improve their therapeutic efficacy while minimizing potential toxicity [19].

In the present study, PLGANP loaded with PSE were formulated and characterized to evaluate their antioxidant and antidiabetic potential. The in-vitro antioxidant and phytochemical properties of PSENP were investigated alongside its in-vivo antihyperglycemic, antioxidant, and anti-inflammatory activities in an alloxan-induced Type 1-like diabetic rat model. Furthermore, in-silico molecular docking and ADMET analyses were performed to explore possible molecular interactions and predict drug-likeness profiles of the identified phytoconstituents [20]. This integrated approach was designed to investigate the therapeutic potential of PSENP as a nano-enabled strategy for oxidative stress-associated experimental diabetes management.

## Materials and methodology

### Chemicals and instruments

Poly (lactic-co-glycolic acid) (PLGA) and polyvinyl alcohol (PVA) was procured Sigma-Aldrich (St. Louis, MO, USA), Dichloromethane, ethanol, ethyl acetate, and methanol were purchased from Merck (Germany). Standard phenolic and flavonoid compounds, including quercetin, gallic acid, caffeic acid, and syringic acid, were obtained from Sigma-Aldrich for comparative HPLC analysis and in-silico studies. Diagnostic kits for the estimation of glucose, insulin, albumin, globulin, lipid profile, liver and kidney function markers, oxidative stress biomarkers, and inflammatory cytokines (TNF-α, IL-6) were sourced from Thermo Fisher Scientific (USA) and QCA Diagnostics (Spain).

The experimental analyses were performed by a high-performance liquid chromatography (HPLC) scheme (Shimadzu, Japan) prepared with a Shim-Pack C18 column, which was utilized for the quantification of individual phytochemical constituents. Zeta potential and particle size measurements of nanoparticles were performed using a Zeta sizer Nano ZS (Malvern Instruments, UK). The UV–Visible spectrophotometer (UV-1601, Shimadzu, Japan) was utilized for the quantification of total phenolic content, total flavonoid content, and various *in-vitro* antioxidant assays. Fourier Transform Infrared (FTIR) spectroscopy was shown by a Bruker Alpha spectrometer to identify functional groups in the nanoparticles. Freeze-drying of the nanoparticle suspensions was carried out using a lyophilizer (Alpha 1–4 LSC, Christ, Germany). Histopathological assessments were conducted using a standard light microscope after tissue processing and hematoxylin and eosin (H&E) staining. All data analyses were done by using GraphPad Prism (version 8.4.2).

### Sample of plant material

*Persea americana* seeds were collected from a local market of Faisalabad. The plant species was botanically authenticated by the Department of Botany, University of Agriculture Faisalabad (UAF), and a voucher specimen was deposited under the reference number UAF Voucher No. 2511-3-024. The collection and use of plant material were conducted in accordance with national and institutional ethical guidelines and regulations.

### Preparation of *Persea americana* seed extract

Fresh *Persea americana* seeds were washed, cut into smaller pieces, and air-dried in a shaded area for five days to prevent degradation of heat-sensitive compounds. Once fully dried, the seeds were ground into a fine powder using an electric blender and stored in a sealed, light-protected container until further use. For extraction, 30 grams of the powdered seed material was soaked in distilled water (500 ml) and allowed to shake for 48 hours at room temperature in an orbital shaker of voltage 100–240 V (Robus Technologies; RTSK-0300 Catalogue no. 832210210288). The resulting mixture was then filtered, and the aqueous extract was concentrated using a hot air oven to obtain a final yield of approximately 20 grams. This procedure was conducted following the procedure outlined by Ojo et al. (2022), with minor amendments.

### Preparation of *Persea americana* seed extract-loaded PLGA nanoparticles

To prepare the nanoparticles, 50 mg of PLGA was first dissolved in 0.5 mL of dichloromethane. Separately, 5 mg of PSE was dissolved in 200 mL of ethanol. Both solutions were then combined in 1.3 mL of ethyl acetate at room temperature to initiate emulsification. This mixture was added dropwise to 10 mL of 0.5% PVA aqueous solution and emulsified using low-frequency ultrasonication at 50% amplitude for 10 minutes to form a stable oil-in-water emulsion.

Following emulsification, the organic solvents were rapidly evaporated under vacuum at 37 °C for 5 minutes. The nanoparticles were then isolated by centrifugation, and the supernatant was carefully removed. The resulting nanoformulation was cooled and subjected to freeze-drying using a lyophilizer (Christ, Germany/Alpha 1–4LSC) to obtain a dry nanoparticle formulation following the method described by Manayi *et al.* (2022) with slide modifications and stored in air tight container for further use [21].

### *In-vitro* study

#### Zeta potential and particle size analysis.

The particle size distribution and zeta potential of the PLGANP and PSENP were measured using a Zeta sizer Nano ZS (Malvern Instruments, UK). All measurements were conducted in triplicate at 25 °C to ensure accuracy and dispersion characteristics of the nanoparticle formulation [22].

#### Fourier Transform Infrared (FTIR) spectroscopy.

FTIR spectroscopy was done via PerkinElmer Spectrum FT-IR Spectrometer equipped with an ATR accessory over the spectral range of 4000–400 cm^-1^, to identify the functional groups and chemical interactions present in both the PSE and the PLGA nanoparticles. The blank PLGANP and PSENP samples were scanned to detect characteristic peaks and assess any potential interactions or structural modifications [23].

#### Transmission Electron Microscopy (TEM).

The morphology and surface characteristics of blank PLGANP and PSENP were examined using transmission electron microscopy (TEM). A drop of freshly prepared nanoparticle suspension was placed onto a carbon-coated copper grid and allowed to adsorb for 30 minutes. Excess liquid was removed using filter paper, followed by air drying at room temperature.

The samples were then observed under a transmission electron microscope (JEOL JEM-1010, Japan) operated at an appropriate accelerating voltage. The particle morphology, size distribution, and degree of aggregation were analyzed from the obtained micrographs [24].

### Colloidal Stability test

Turbidity assay was performed on the blank PLGANP and PSENP via UV–Visible spectrophotometer to evaluate nanoparticle aggregation behavior and colloidal stability. The nanoparticle suspensions were prepared in distilled water and stored at 4°C throughout the study period. The optical density of nanoparticle suspensions was measured at 600 nm at 0 h, 24 h, and 7 days. The absorbance was considered as marker to indicate the nanoparticle aggregation potency. The stable absorbance values over time indicated colloidal stability. All measurements were determined in triplicate.

### Chromatographic profiling of bioactive compounds

High-performance liquid chromatography (HPLC) was used to identify and quantify phenolic and flavonoid constituents in the PSE. The analysis was carried out using a Shimadzu HPLC system (Japan), equipped with a Shim-Pack C18 column (5 µm, 250 mm × 4.6 mm) and maintained at 30 °C. A 20 µL volume of the sample was injected for each run.

The mobile phase consisted of acetic acid and acetonitrile, delivered at a flow rate of 1 mL/min. Detection was performed using a UV/Vis detector set at 280 nm following the method described by Ong *et al.* (2022) with minor modification. Chromatographic data were recorded using the Chromera software system. Identification and quantification of compounds were based on comparison of retention times and spectral characteristics with standard phenolic and flavonoid compounds [25].

All reference standards for phenolic acids and flavonoids were procured from Sigma Chemical Co. (St. Louis, MO, USA), while other analytical-grade reagents and solvents were obtained from Merck (Germany). This high-resolution chromatographic technique allowed for accurate separation and quantification of the bioactive compounds present in the extract [26].

### Estimation of total phenolic and flavonoid contents

For the estimation of total phenolic content (TPC) in PSE and PSENP, 15 mg of each sample was placed into a clean test tube and combined with 500 µL of distilled water. Subsequently, 0.125 mL of Folin–Ciocalteu reagent ((FCR) was added, and the mixture was permissible to react for 6 min. Subsequently, a volume of 1.25 mL sodium carbonate solution (7%) was putted, and the total volume was adjusted to 3 mL with distilled water. The mixture was hatched at room temperature for 90 min to complete the reaction. Absorbance was measured at 760 nm using a UV-Visible spectrophotometer. The phenolic content was calculated based on a gallic acid calibration curve and results were stated in mg gallic acid equivalents (GAE) per gram of sample [27].

For the determination of total flavonoid content (TFC), 15 mg of the extract was dissolved in 1.25 mL of distilled water. Subsequently, 75 µL of a 5% sodium nitrite solution was added, and after a 6-minute incubation period, 0.150 mL of an aluminum chloride (10%) mixture was presented. The solution was then adjusted to 2.5 mL using distilled water, and the contents were carefully mixed. Absorbance was measured promptly at 510 nm utilizing a UV-Visible spectrophotometer. Catechin was applied as the standard, and a calibration curve was constructed using concentrations ranging from 0.2 to 0.9 mg/mL in methanol [28].

### DPPH radical scavenging activity

The antioxidant capacity of PSE and PSENP was evaluated using the DPPH free radical scavenging assay, as described by Yurtsever *et al.* (2024) [29] with slide modifications. For sample preparation, 15 mg of extract was dissolved in 10 mL of 80% methanol and allowed to stand for 2 hours.

A DPPH working solution was prepared by dissolving 4 mg of DPPH in 100 mL of methanol. In this assay, 2 mL of the DPPH solution was combined with 50 µL of the sample extract, followed by thorough mixing. The reaction mixture was then incubated in the dark at room temperature for 30 minutes. Subsequently, the reduction in absorbance was recorded at 515 nm using a UV–Visible spectrophotometer, with acarbose serving as the reference antioxidant.

### Alpha-glucosidase inhibition assay

The inhibitory activities of PSE and PSENP against alpha-glucosidase were evaluated following to the method described by Nguelefack et al. (2020), with minor adjustments. To prepare the enzyme solution, 1 mg of alpha-glucosidase was liquified in 100 mL of phosphate buffer (pH 6.8) containing 200 mg of bovine serum albumin (BSA) to enhance enzyme stability [30].

For the assay, 15 mg of the test sample was mixed with 490 µL of phosphate buffer (pH 6.8) and 250 µL of 5 mM *p*-nitrophenyl α-D-glucopyranoside, the substrate. The mixture was pre-incubated at 37 °C for 15 min, after which the reaction was terminated by the addition of 2 mL of sodium carbonate (Na_2_CO_3_) (200 mM). The absorbance was then recorded at 400 nm using a UV–Visible spectrophotometer. Acarbose, a recognized alpha-glucosidase inhibitor, served as the positive control for comparison.

### Determination of encapsulation efficiency and drug loading

The encapsulation efficiency (%EE) and drug loading (%DL) of the PLGA nanoparticles were determined by indirect quantification. After centrifugation of the nanoparticle suspension at 15,000 rpm for 30 min, the supernatant was collected and analyzed for unencapsulated extract using UV–Vis spectrophotometry at 280 nm. The %EE and %DL were calculated according to the following equations:

EE(%)=\frac{W_{initial\ drug}−W_{free\ drug}}{W_{initial\ drug}}\times100


$$\begin{array}{c} DL(\%)=\backslash frac\{W\_\{encapsulated\backslash \;drug\}\}\{W\_\{nanoparticles\}\}\backslash times\text{1}\text{0}\text{0} \end{array}$$

where, W_initial drug_ is the total amount of drug initially used during nanoparticle preparation, W_free_ drug is the amount of unencapsulated drug present in the supernatant, W_encapsulated drug_ is the amount of drug entrapped within the nanoparticles, and W_nanoparticles_ is the total weight of recovered nanoparticles.

### In-Silico analysis

#### Identification and screening of bioactive compounds.

Bioactive compounds identified through HPLC analysis of *Persea americana* seed extract were selected for in-silico evaluation based on their drug-likeness potential. The chemical name, ID and SMILES notation of these natural inhibitors were retrieved from PubChem, a freely available database maintained by the National Institutes of Health (NIH). PubChem serves as a vital resource for various stages of drug discovery, including lead identification, compound profiling, and structure-activity relationship studies.

#### Virtual screening and molecular docking.

To assess the binding affinity of the selected phytochemicals toward the target protein (alpha-glucosidase), virtual screening was conducted using PyRx, an open-source tool commonly used in molecular docking studies. The receptor protein was first prepared and converted to PDBQT format. Ligands obtained from PubChem were energy-minimized and similarly converted to PDBQT files. Docking simulations were then performed to predict the binding interactions and estimate binding energies. Compounds showing the strongest interactions were considered potential inhibitors.

#### ADMET prediction.

The pharmacokinetic and toxicity profiles of the docked compounds were evaluated using the PreADMET online tool. This computational approach enables early prediction of absorption, distribution, metabolism, excretion, and toxicity (ADMET) properties, helping to filter out unsuitable drug candidates before costly experimental validation. This step enhances the efficiency of the drug development pipeline by focusing only on compounds with acceptable safety and efficacy profiles.

#### Drug-likeness evaluation using lipinski’s rule.

To assess drug-likeness, the selected ligands were further analyzed using the Molinspiration tool, which calculates key molecular properties and bioactivity scores. Lipinski’s Rule of Five was applied to determine oral bioavailability. According to this rule, a compound is more likely to be orally active if it has no more than five hydrogen bond donors, no more than ten hydrogen bond acceptors, a molecular weight under 500 Da, and a Log P value below 5. These criteria help predict the pharmacokinetic behavior and guide compound selection for further *in-vitro* or *in-vivo* studies.

### *In-vivo* analysis

#### Experimental animals.

A total of 40 healthy adult male albino rats were obtained from the Animal House Facility of the Institute of Physiology and Pharmacology, University of Agriculture, Faisalabad. Animals were housed under standard laboratory conditions and allowed to acclimatize for one week prior to the commencement of the study. During acclimatization, rats were provided with standard commercial feed and water ad libitum, with regular replacement twice daily.

All experimental procedures were conducted in accordance with the National Institutes of Health Guide for the Care and Use of Laboratory Animals and were approved by the Institutional Bioethics Committee of the University of Agriculture, Faisalabad (Protocol Ref: D. No. 5048/ORIC).

For anesthesia, sodium pentobarbital was administered via intraperitoneal injection at an appropriate anesthetic dose to ensure loss of consciousness and absence of pain during experimental procedures. At the end of the study, animals were humanely euthanized by administering an overdose of sodium pentobarbital (intraperitoneal), in accordance with accepted euthanasia guidelines, prior to sample collection.

Throughout the study, all possible measures were taken to minimize pain, distress, and discomfort. Animals were monitored twice daily during diabetes induction and disease progression, and once daily during the treatment phase. Any animal showing signs of distress, illness, or abnormal behavior received additional observation and supportive care when required. Handling was performed gently and only by trained personnel.

#### Induction of diabetes.

After acclimatization, rats were randomly assigned into five groups (n = 8 per group) [31].

1. Group A: Normal control (received food and water)
2. Group B: Diabetic control (PBS p.o.)
3. Group C: Diabetic + metformin (31.5 mg/kg body weight p.o.)
4. Group D: Diabetic + PSE (106.5 mg/kg p.o.)
5. Group E: Diabetic + PSENP (70.5 mg/kg p.o.)

Diabetes was induced in all groups except the normal control by a single intraperitoneal injection of alloxan monohydrate (130 mg/kg body weight) in cold saline [32]. Six hours’ post-injection, rats were given 20% glucose orally to prevent fatal hypoglycemia. After 72 hours, fasting blood glucose was measured. Rats with glucose levels exceeding 180 mg/dL were considered diabetic. All treatments were administered once daily via oral gavage using a gastric feeding tube throughout the experimental period of 21 days.

#### Monitoring of metabolic parameters: fasting glucose and weight.

Fasting blood glucose levels were regularly monitored throughout the study to evaluate the glycemic response of rats to both disease induction and treatment. Measurements were taken prior to alloxan administration, after diabetes induction, and then weekly during the 21-day treatment period. Blood samples were collected from the tail vein, and glucose concentrations were determined using a digital glucometer. In parallel, the body weight of each animal was recorded weekly using a digital weighing balance (G&G® Model No. JJ2244BC) to observe any treatment-related metabolic effects and to assess general health status.

#### Monitoring of glycemic biomarkers.

Serum glucose levels were determined using a commercial diagnostic kit (Sigma-Aldrich, Cat# MAK181), following the manufacturer’s protocol. Insulin levels were measured using a Thermo Fisher ELISA kit (Cat# ERIN), with absorbance recorded at 450 nm. These biomarkers were evaluated to determine the impact of the treatments on glycemic control and insulin responsiveness in diabetic rats.

#### Monitoring of inflammatory biomarkers.

To evaluate the systemic inflammatory response, serum levels of albumin and globulin were measured using QCA diagnostic kits (Cat# 99725 and 998690, respectively). These proteins serve as reliable indicators of protein metabolism, hepatic performance, and underlying inflammatory processes. In addition, key pro-inflammatory cytokines, interleukin-6 (IL-6) and tumor necrosis factor-alpha (TNF-α)—were quantified using standard sandwich ELISA protocols (Thermo Fisher, Cat# ERA31RB and ERA56RB) [32]. Monitoring these markers provided valuable insight into how the treatments influenced inflammation in diabetic conditions.

### Determination of oxidative stress biomarkers

Oxidative stress and antioxidant capacity were measured by measuring total antioxidant capacity (TAC) and total oxidant status (TOS) using validated commercial assay kits, TAC with Sigma-Aldrich (Cat# MAK187) and TOS with Thermo Fisher (Cat# EEA027) [33]. Superoxide dismutase (SOD) activity, a key antioxidant enzyme, was assessed by observing its ability to prevent the breakdown of nitro blue tetrazolium (NBT), with results read at 560 nm. Catalase (CAT) activity, another important antioxidant marker, was measured by tracking the rate at which it broke down hydrogen peroxide at 240 nm using a phosphate buffer system following the method of Mandlik *et al.* (2022). These tests offered valuable insight into the oxidative stress levels in the animals and helped demonstrate how well the treatments supported the body’s natural antioxidant defences [34].

### Lipid profile assessment

To evaluate the impact of treatment on lipid metabolism, blood samples were collected from each animal at the end of the study. After clotting, the samples were centrifuged at 3000 rpm for 15 minutes to separate the serum. Serum lipid parameters were then analysed to assess cardiovascular risk factors often associated with diabetes. Total cholesterol (TC), triglycerides (TG), and high-density lipoprotein (HDL) were measured using the established methods of Richmond (1973), Fassati and Principe (1982), and Gordon (1977), respectively. Low-density lipoprotein (LDL) was calculated using the Friedewald equation as described by Hatch and Lees (1968) [35].

### Histopathological examination

The pancreas was carefully removed and preserved in 10% neutral buffered formalin to maintain tissue structure. Following fixation, the samples were embedded in paraffin, sectioned into thin slices (4–6 µm), and stained with hematoxylin and eosin (H&E) for microscopic examination under a light microscope [36]. This analysis helped visualize changes in pancreatic architecture, particularly in the islets of Langerhans, allowing assessment of tissue recovery following treatment.

### Toxicological and safety assessment

To ensure that the treatment was biologically safe and did not harm vital metabolic and excretory organs (liver and kidney), a comprehensive toxicological evaluation was carried out alongside therapeutic assessments [37]. The liver and kidney tissues were carefully examined for any structural changes or signs of toxicity using standard histological methods.

In addition to tissue examination, standard liver and renal function tests were performed to assess systemic toxicity. Liver enzyme levels including alanine aminotransferase (ALT), aspartate aminotransferase (AST), and alkaline phosphatase (ALP)—were measured using QCA diagnostic kits (Ref. 993615, 998003, and 996265, respectively). Renal function markers, blood urea nitrogen (BUN) and serum creatinine, were also determined using appropriate diagnostic protocols. These evaluations were essential to confirm that the treatment did not compromise the health of vital organs, ensuring its safety for potential therapeutic use.

### Statistical analysis

Data was analysed using graph pad prism (version 8). One-way analysis of variance (ANOVA) was used to compare differences among treatment groups for most parameters. For repeated blood glucose measurements over time, two-way ANOVA was applied to assess both group and time effects. A p-value of less than 0.05 was considered statistically significant.

## Results

### Zeta size and polydispersity index of nanoformulation

Dynamic light scattering analysis indicated that the blank PLGANP had an average hydrodynamic diameter of 312.6 nm, as shown in Fig 1A, while in Fig 1B that of PSENP increased to 471.2 nm, indicating successful encapsulation of the extract. The increase in particle size following extract loading may be attributed to the incorporation of phytoconstituents within the polymeric matrix. The polydispersity index (PDI) values were recorded as 0.251 for PLGANP and 0.1768 for PSENP, suggesting a relatively uniform particle distribution, and acceptable formulation stability. This increase in size upon loading and the acceptable PDI confirm the formation of well-dispersed, nanoscale drug carriers suitable for biological applications.

**Fig 1:**
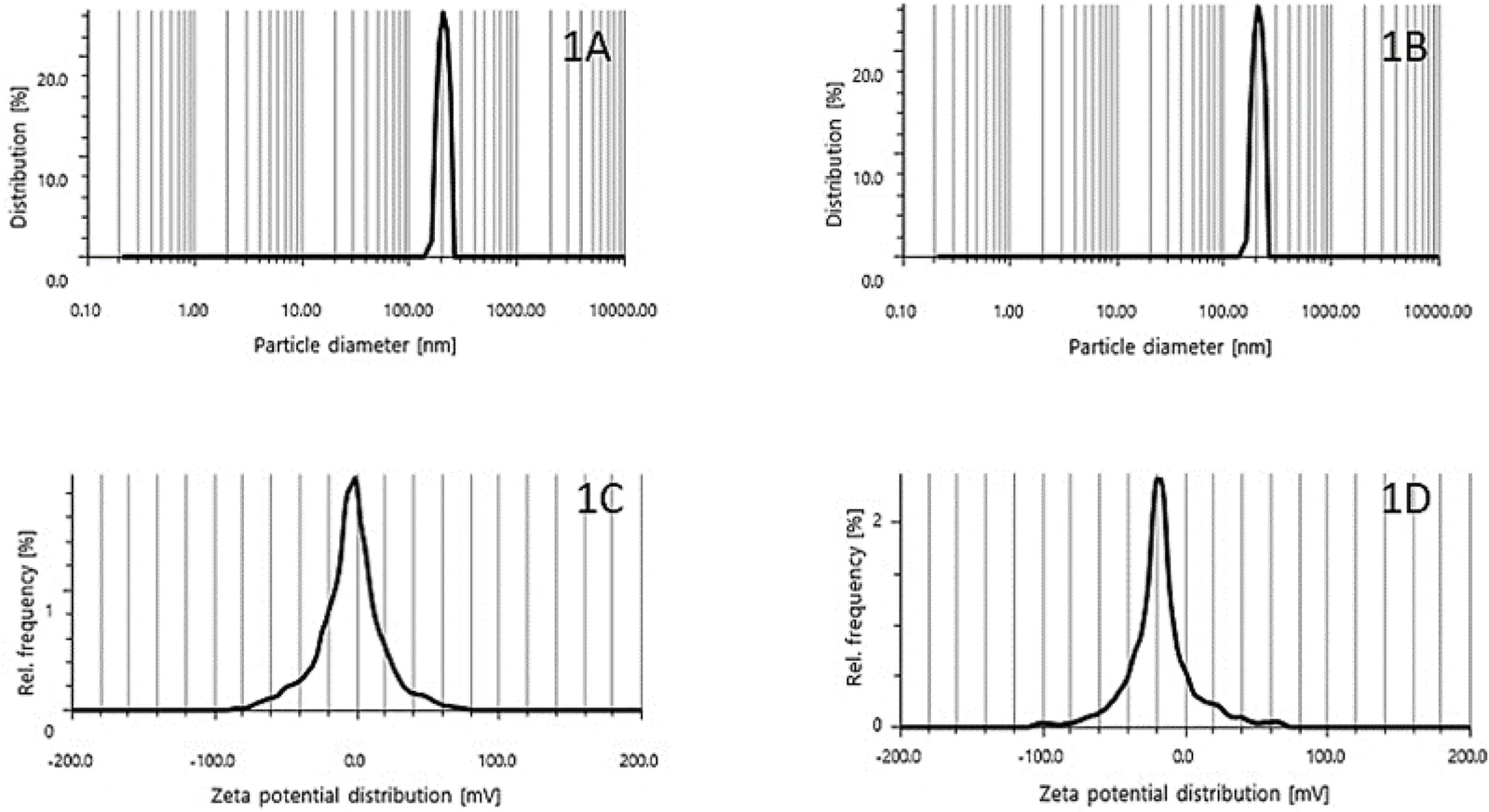
Particle size distribution and zeta potential analysis of PLGANP and PSENP (1A) Zeta size of PLGA nanoparticles; (1B) Zeta size of PSENP; (1C) Zeta potential of PLGA nanoparticles; (1D) Zeta potential of PSENP.

The corresponding peak zeta potential value was –10.5 mV for blank PLGANP in Fig 1C and –16.8 mV for PSENP in Fig 1D, indicating a shift toward increased surface charge after drug loading. The enhanced negative surface charge of PSENP suggests improved colloidal stability and reduced risk of nanoparticle aggregation, making the formulation more suitable for biomedical applications.

### Transmission Electron Microscopy (TEM) analysis

The morphology and structural characteristics of blank PLGANP and PSENP were examined using TEM, as shown in Fig 2.

**Fig 2:**
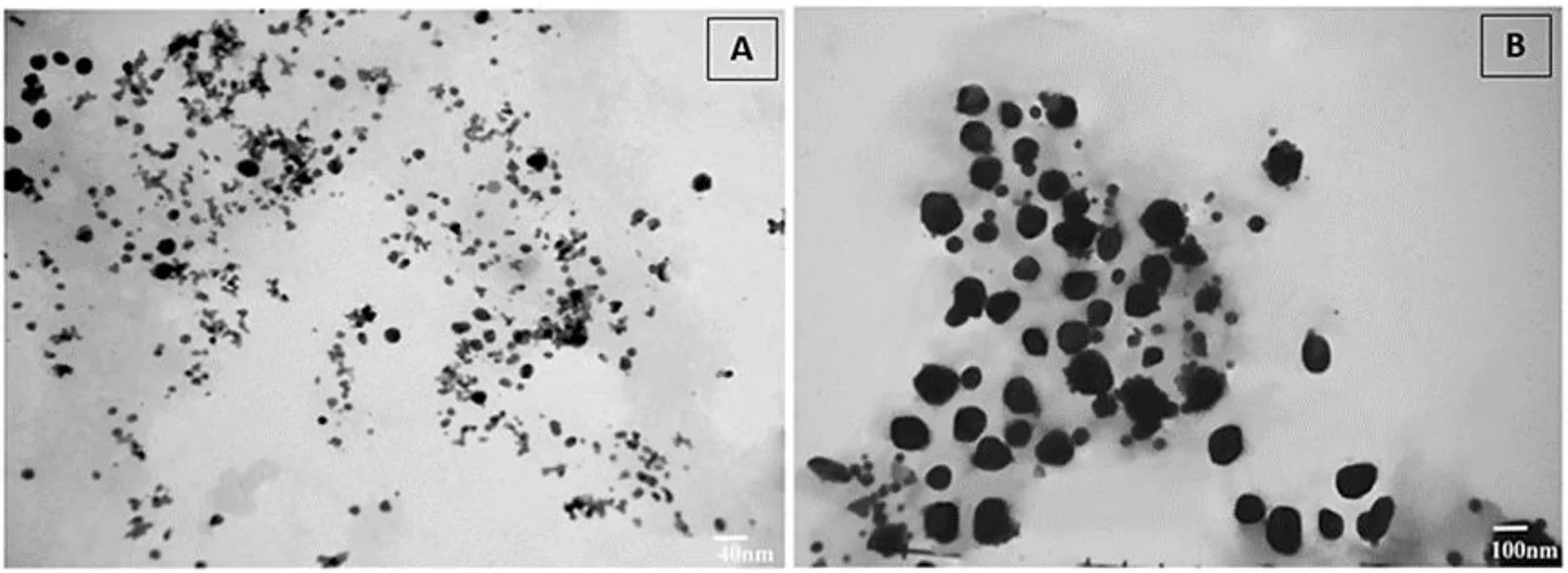
Transmission electron microscopy (TEM) images of blank PLGANP and PSENP (A) PLGANP showing a predominantly spherical morphology with uniform distribution and an average particle size of 40 nm. (B) PSENP exhibiting a spherical morphology with an average particle size of approximately 100 nm.

As illustrated in Fig 2A the blank PLGANP exhibited a predominantly spherical morphology with a relatively uniform size distribution. The nanoparticles appeared well dispersed throughout the field with minimal aggregation, indicating successful nanoparticle formation and good colloidal stability. The average particle size of PLGANP was approximately 40 nm.

Following encapsulation of PSE, the PSENP formulation retained its characteristic spherical morphology (Fig 2B). However, a moderate increase in particle size was observed, with an average diameter of approximately 100 nm. In addition, a slight increase in particle clustering was observed in some regions compared to the PLGANP. This mild aggregation may be attributed to particle interactions during sample preparation and solvent evaporation on the TEM grid. Despite the presence of mild clustering, individual nanoparticles remained clearly distinguishable, confirming the successful fabrication of the extract-loaded PLGA nanoparticles.

Overall, TEM analysis demonstrated that incorporation of PSE did not markedly alter the morphological characteristics of the PLGANP, and both formulations maintained a predominantly spherical nanoscale structure. The observed increase in particle size following extract loading provides further evidence of successful incorporation of *Persea americana* seed extract within the PLGA matrix.

### Turbidity analysis

The colloidal stability of PLGANP and PSENP was assessed by monitoring turbidity at 600 nm over a period of 7 days. As shown in Fig 3, both formulations exhibited only minor changes in absorbance throughout the study period.

**Fig 3:**
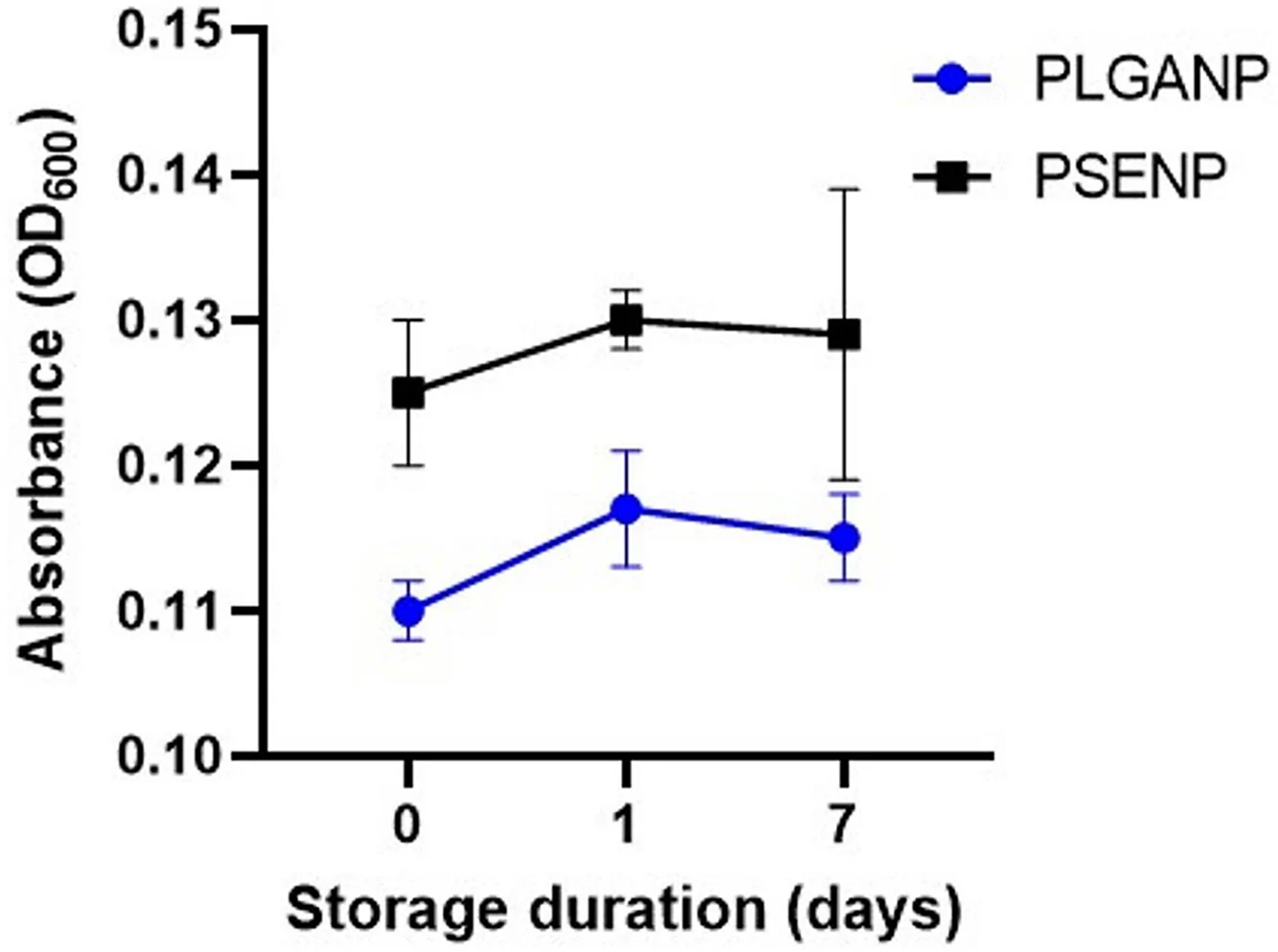
Stability assessment of PLGANP and PSENP by turbidity analysis. Turbidity profiles of blank PLGANP and PSENP. Data are presented as mean ± standard deviation (SD) (n = 3).

The PLGANP showed absorbance value of 0.110 ± 0.002 at 0 h, 0.117 ± 0.004 at 24 hour and 0.115 ± 0.003 after 7 days, hence indicating relatively constant values throughout study duration. Similarly, the PSENP showed negligible change in turbidity, with absorbance values of 0.125 ± 0.005, 0.131 ± 0.002, and 0.126 ± 0.001 at 0 h, 24 h, and 7 days, respectively.

No statistically significant increase in optical density was observed for either formulation during storage, indicating minimal particle aggregation and sedimentation. Furthermore, the turbidity profile of the extract-loaded nanoparticles was comparable to that of blank PLGA nanoparticles, suggesting that incorporation of PSE did not adversely affect nanoparticle stability. These findings demonstrate good colloidal stability of the nanoparticle formulations under the experimental conditions.

### HPLC analysis of PSE

The HPLC analysis of PSE revealed a diverse profile of bioactive polyphenolic and flavonoid compounds. Notably, 1-caffeoylquinic acid was identified with a retention time of 3.533 minutes, while quercetin-3-O-glucoside and quercetin appeared at 5.313 and 6.400 minutes, respectively, indicating the presence of potent flavonoids. Syringic acid, a known antioxidant phenolic acid, was eluted at 21.547 minutes, whereas epicatechin, a statistically significant flavanol, was detected at 27.633 minutes. The detection of these compounds with distinct voltages and retention times confirms the rich phytochemical composition of the extract. This diversity of phenolic and flavonoid constituents contributes significantly to the antioxidant and therapeutic properties of PSE, validating its potential in pharmaceutical applications. These findings are detailed in Table 1, accompanied by chromatogram (Fig 4) indicating the respective retention times of each compound.

**Table 1:** Peak number, Voltage, Retention time, and molecular formula and peak number of *P. americana* seed extract phytochemical compounds.

Peak No.	Compound name	Voltage (m/V)	Retention time (min)	Molecular formula
3	1-caffeoylquinic acid	6.122	3.533	C_16_H_18_O_9_
5	Quercetin-3-o-glucoside	8.100	5.313	C_15_H_14_O_6_
7	Quercetin	36.233	6.400	C_15_H_10_O_7_
20	Syringic acid	19.452	21.547	C_9_H_10_0_5_
24	Epicatechin	5.891	27.633	C_15_H_14_O_6_

**Fig 4:**
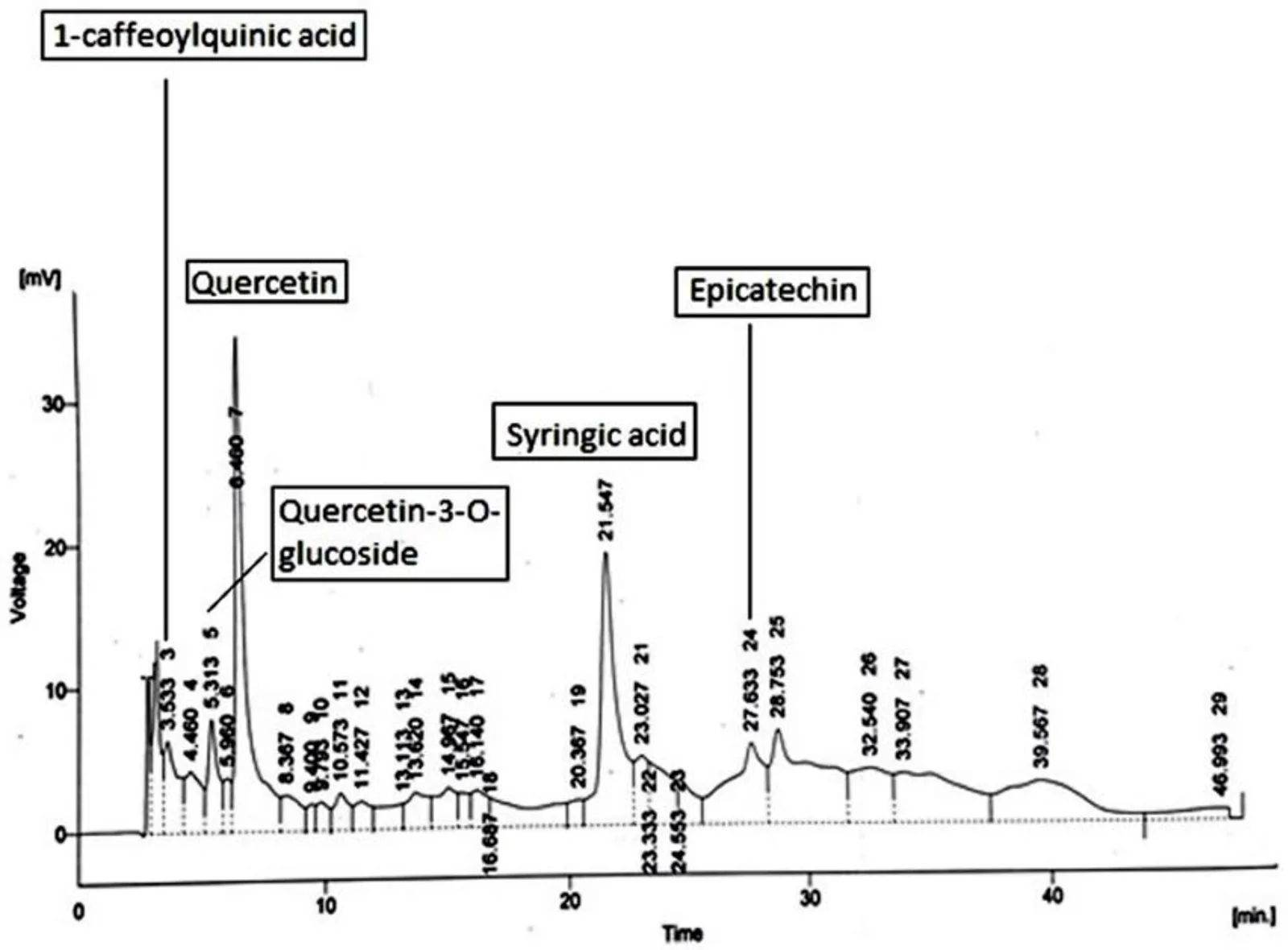
High Performance Liquid Chromatogram of *P. americana* seed extract.

### FTIR spectral analysis

The FTIR spectra of blank PLGANP and PSENP revealed characteristic absorption bands confirming successful nanoparticle formation and phytoconstituent encapsulation (Fig 5).

**Fig 5:**
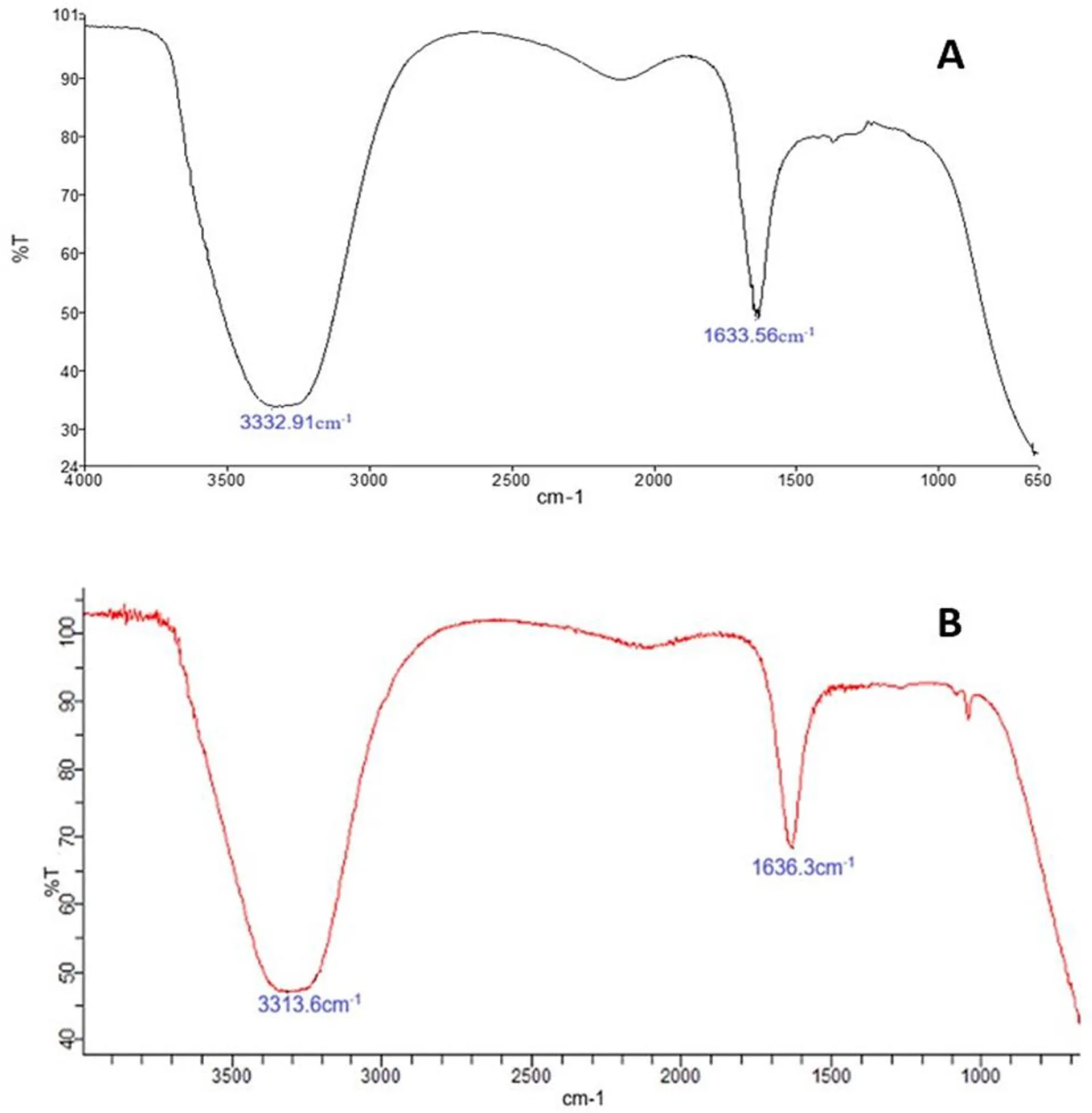
FTIR spectral analysis of PLGANP and PSENP. (A) FTIR spectrum of blank PLGANP, showing characteristic absorption bands corresponding to O–H stretching (~3332.91 cm^-1^) and C = O stretching (~1633.56 cm^-1^), confirming the structural integrity of the PLGA polymer. (B) FTIR spectrum of PSENP exhibiting shifted O–H (~3313.6 cm^-1^) and C = O (~1638.3 cm^-1^) stretching bands, indicating successful encapsulation of phytoconstituents and intermolecular interactions within the polymeric matrix.

In PLGANP, a broad absorption band was observed at 3332.91 cm^-1^, corresponding to O–H stretching vibrations, which may be attributed to terminal hydroxyl groups and adsorbed moisture. Another prominent peak at 1633.56 cm^-1^ was assigned to C = O stretching vibrations, characteristic of ester linkages present in the PLGA polymer backbone (Fig 5A).

In contrast, PSENP exhibited a shifted and broadened O–H stretching band at 3313.6 cm^-1^, indicating possible hydrogen bonding interactions between PLGA and phytochemical constituents of the seed extract. The carbonyl-related absorption band was observed at 1638.3 cm^-1^, suggesting subtle changes in the microenvironment of ester/carbonyl groups due to successful loading of bioactive compounds (Fig 5B).

The minor shifts in both O–H and C = O stretching frequencies between PLGANP and PSENP confirm intermolecular interactions rather than chemical degradation, indicating that the structural integrity of PLGA was preserved during formulation. Furthermore, the presence of hydroxyl- and carbonyl-associated bands supports the incorporation of phenolic and flavonoid-rich phytoconstituents from *Persea americana* seed extract.

Overall, FTIR analysis provides strong evidence of successful encapsulation of plant bioactives within PLGA nanoparticles, with maintained polymer stability and potential enhancement of hydrogen-bond-driven stability, which may contribute to improved antioxidant and therapeutic performance.

### Total phenolic content and total flavonoid content determination

The determination of Total Phenolic Content (TPC) and Total Flavonoid Content (TFC) between PSE and PSENP reveals notable variations in their phytochemical composition. As shown in Fig 6, the TPC of PSENP was slightly higher (~285 mg GAE/g) compared to that of PSE (~270 mg GAE/g), and this reduction was statistically significant (p < 0.001). The decline may reflect partial degradation or inefficient encapsulation of certain phenolic compounds during the nanoparticle preparation process.

**Fig 6:**
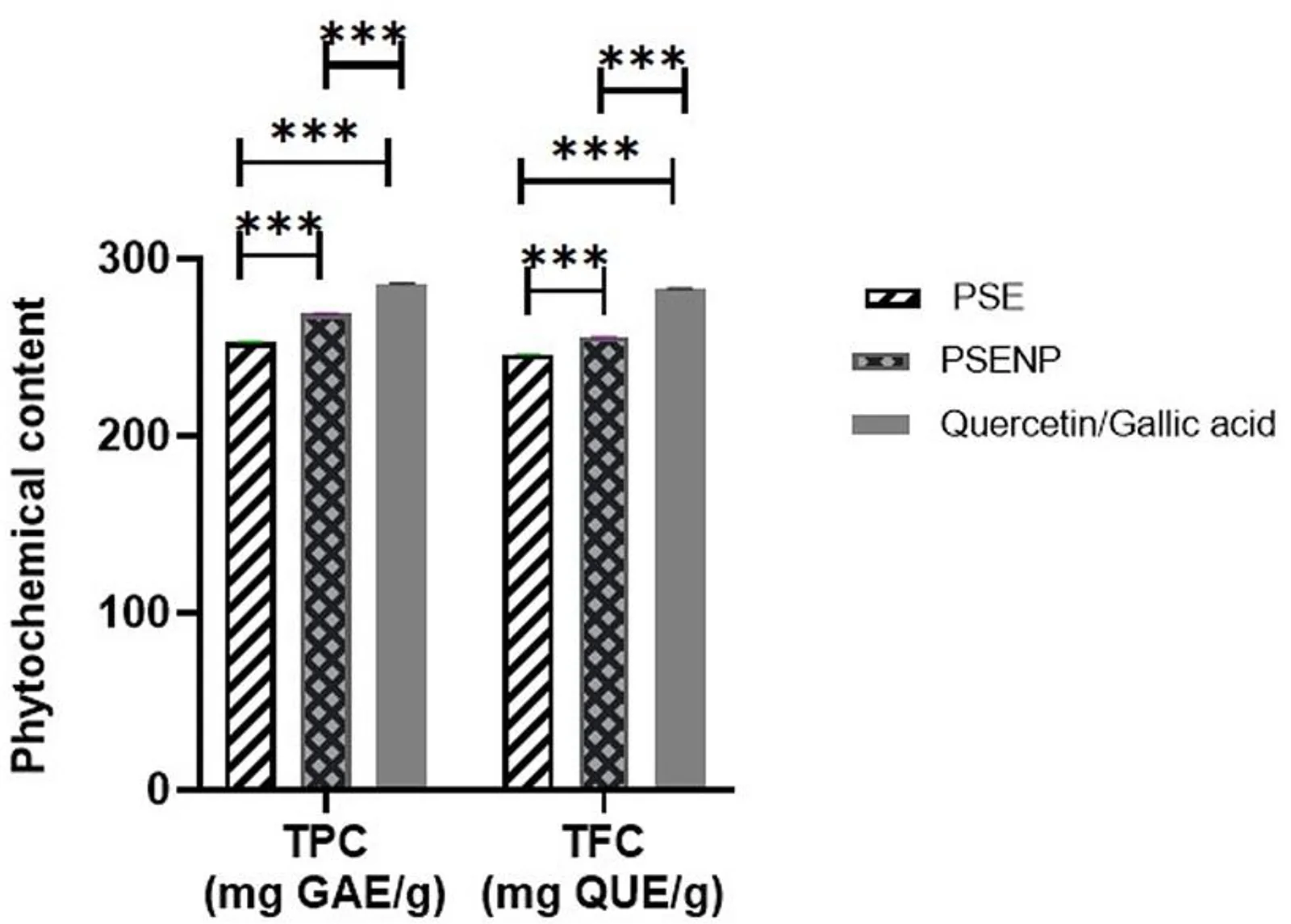
Comparative analysis of total phenolic content (TPC) and total flavonoid content (TFC) in PSE and PSENP, The comparison showing improved flavonoid retention post-encapsulation. Data are represented as mean±SD (n = 3), ***p < 0.001 (one –way ANOVA followed by Tukey’s post hoc test).

Conversely, a marked enhancement in TFC was observed in PSENP (~265 mg QUE/g) compared to the native extract (~250 mg QUE/g), with the difference being statistically significant (p < 0.001). This increase suggests that encapsulation may offer improved stabilization or protection of flavonoid molecules, potentially reducing their degradation and enhancing their retention.

These findings imply that PSENP exhibited a slight increase in total phenolic content (TPC) and total flavonoid content (TFC) compared with the crude extract, indicating improved retention of phytoconstituents following PLGA encapsulation.

### Alpha-Glucosidase Inhibition and DPPH Radical Scavenging Activity

In Fig 7, the comparative graphical analysis underscores the enhanced biological efficacy of PSE, its nanoformulation, i.e., PSENP, and the standard antidiabetic agent acarbose in inhibiting α-glucosidase activity and scavenging DPPH free radicals. In the α-glucosidase inhibition assay, PSENP exhibited significantly higher inhibitory activity than both the crude extract and acarbose (p < 0.001), reflecting its improved potential for modulating postprandial glucose levels.

**Fig 7:**
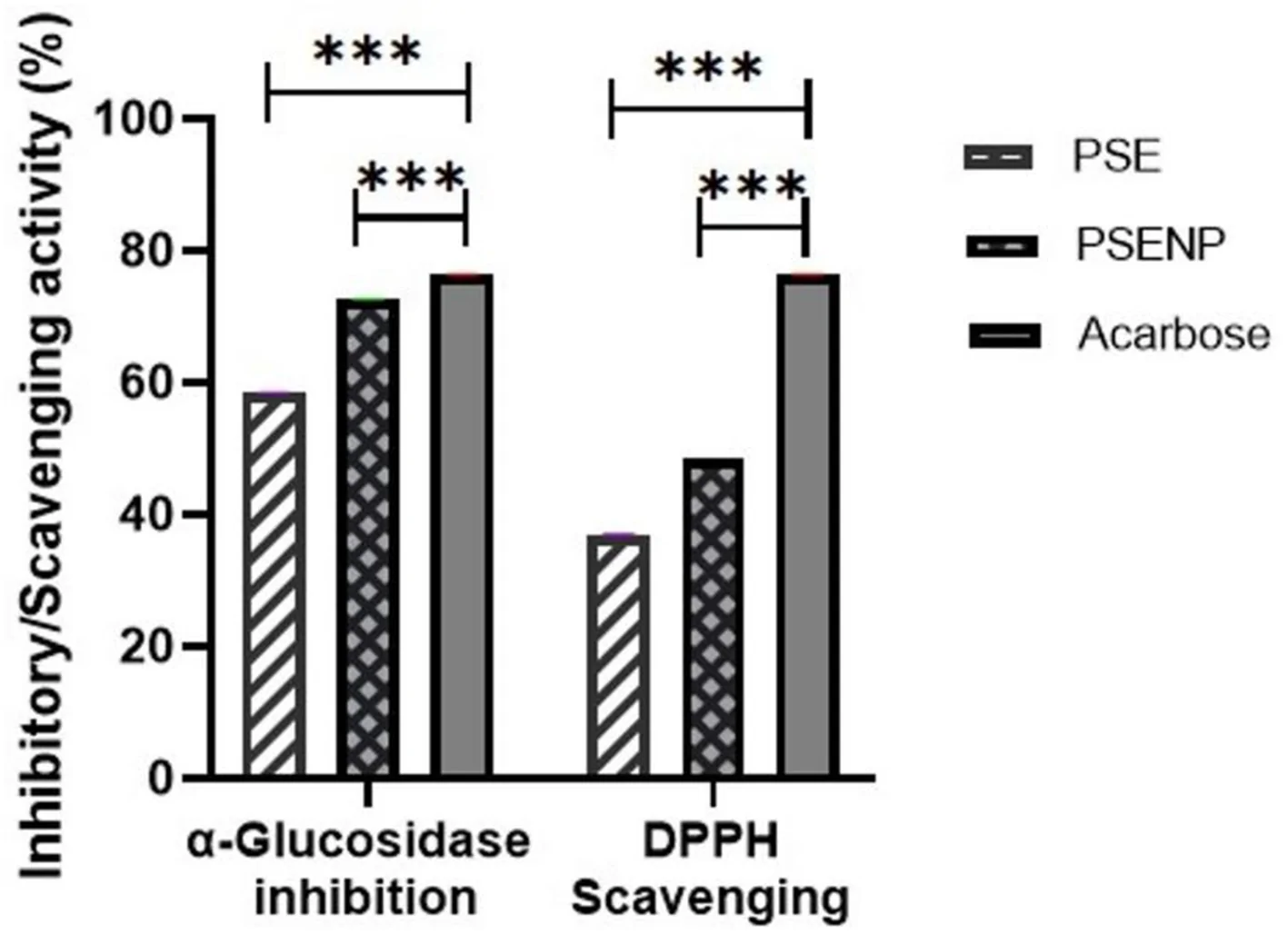
Inhibitory effects of PSE, PSENP, and acarbose on α-glucosidase and DPPH activity. Comparative assessment indicated that PSENP has improved α-glucosidase and DPPH radical scavenging activity as compared to PSE. Data are presented as mean ± SD (n = 3).

Likewise, in the DPPH radical scavenging assay-a widely used indicator of antioxidant capacity PSENP again outperformed both PSE and the standard compound, with a statistically significant increase in activity (p < 0.001). This enhancement may be attributed to the nanoparticle system’s ability to preserve and deliver active constituents more efficiently, likely due to improved solubility, stability, and bioavailability.

Collectively, these findings highlight the dual therapeutic potential of PSENP, demonstrating its promise as a potent natural agent for managing both oxidative stress and hyperglycemia.

### Encapsulation Efficiency and Drug Loading

The PLGA nanoparticles exhibited an encapsulation efficiency of 82.4 ± 3.2% and a drug loading of 7.6 ± 0.5%. These values demonstrate that the formulation process effectively incorporated the ethanolic *Persea americana* seed extract into the PLGA matrix. The high encapsulation efficiency can be attributed to the hydrophobic nature of the extract constituents, mainly phenolic and fatty acid compounds in which show strong affinity toward the hydrophobic PLGA polymer. This interaction minimizes extract diffusion into the aqueous phase during nanoparticle formation and enhances entrapment within the polymeric core. In Fig 8, ethanolic PSENP drug released within intervals of time, result was statistically significant.

**Fig 8:**
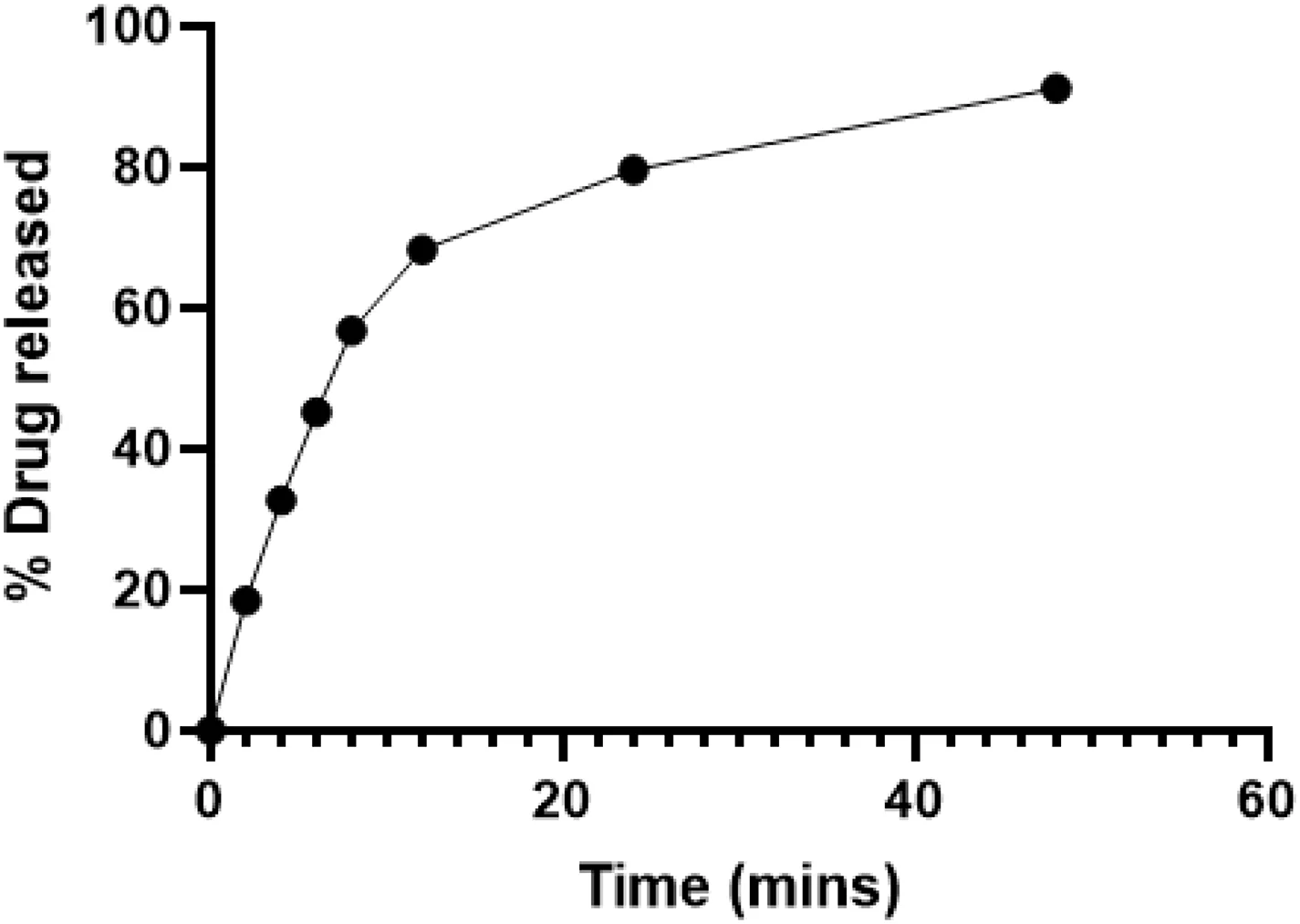
*In vitro* drug release profile of Persea americana seed extract-loaded PLGA nanoparticles (PSENP). Cumulative percentage drug release from PSENP over the indicated time intervals. Data are presented as mean ± SD (n = 3). Statistical significance was determined using a two-tailed test, with *p < 0.05 considered statistically significant.

Furthermore, the use of ethanol as the extraction solvent may have improved compatibility between the extract and PLGA, facilitating uniform dispersion and stable nanoparticle formation. The optimized ratio of polymer to extract also likely contributed to maintaining nanoparticle integrity while maximizing encapsulation.

### *In-silico* analysis

#### Retrieval of natural inhibitors.

A total of five bioactive compounds were successfully identified in *Persea americana* seed extract through HPLC profiling and subsequently validated against current scientific literature for their pharmacological significance. Selection criteria were based on their drug-likeness and predicted biological efficacy. Comprehensive chemical information, including SMILES notation and compound ID, was retrieved from the PubChem database. A summary of these selected phytoconstituents is provided in Table 2.

**Table 2:** Natural inhibitors from *P. americana* seed extract retrieved from PubChem.

Sr. no.	Compound name	Compound ID	SMILE
1	caffeoylquinic acid	10155076	C1[C@H](C([C@@H](CC1(C(=O)O)OC(=O)/C = C/C2 = CC(=C(C = C2)O)O)O)O)O
2	Quercetin-3-o-glucoside	5280804	C1 = CC(=C(C = C1C2=C(C(=O)C3 = C(C = C(C = C3O2)O)O)O[C@H]4[C@@H]([C@H]([C@@H]([C@H](O4)CO)O)O)O)O)O
3	Quercetin	5280343	C1 = CC(=C(C = C1C2=C(C(=O)C3 = C(C = C(C = C3O2)O)O)O)O)O
4	Syringic acid	10742	COC1 = CC(=CC(=C1O)OC)C(=O)O
5	Epicatechin	72276	C1[C@H]([C@H](OC2 = CC(=CC(=C21)O)O)C3 = CC(=C(C = C3)O)O)O

#### Screening of natural inhibitors.

The selected natural inhibitors were virtually screened using PyRx software to evaluate their binding affinities with the alpha-glucosidase target protein (PDB ID: 7JTY) that protein ID, protein sequence and smile as mentioned in Table 3. Molecular docking against protein 7JTY showed that quercetin had the highest binding affinity (–9.8 kcal/mol), followed by quercetin-3-O-glucoside (–9.6 kcal/mol) and 1-caffeoylquinic acid (–9.2 kcal/mol). These flavonoid compounds demonstrated stronger interactions compared to others, suggesting their potential as effective modulators of the target protein. Table 4 shows molecular docking results of natural inhibitors with alpha-glucosidase (PDB ID: 7JTY). Conventional hydrogen bonds were formed with ARG426, unfavorable donor–donor interactions were formed with ARG467, and π-alkyl and π-cation interactions were formed. These comprehensive observations deepen understanding of the binding modes and interactions of these compounds with α-glucosidase inhibitor are reported 2-D structural representation in Fig 9.

**Table 3:** Compound name, protein ID, protein sequence and protein smile are retrieved from PDB.

Sr.no	Compound	Protein ID	P Protein Sequence	SMILE
1	Quercetin	7JTY	MEMEKEFEQIDKSGSWAAIYQDIRHEASDFPCRVAKLPKNKNRNRYRDVSPFDHSRIKLHQEDNDYINASLIKMEEAQRSYILTQGPLPNTCGHFWEMVWEQKSRGVVMLNRVMEKGSLKCAQYWPQKEEKEMIFEDTNLKLTLISEDIKSYYTVRQLELENLTTQETREILHFHYTTWPDFGVPESPASFLNFLFKVRESGSLSPEHGPVVVHSSAGIGRSGTFCLADTCLLLMDKRKDPSSVDIKKVLLEMRKFRMGLIQTADQLRFSYLAVIEGAKFIMGDSSVQDQWKELSHEDLEPPPEHIPPPPRPPKRILEPHN	C1 = CC(=C(C = C1C2=C(C(=O)C3 = C(C = C(C = C3O2)O)O)O)O)O

**Table 4:** Molecular docking results of natural inhibitors with alpha-glucosidase (PDB ID: 7JTY).

Simulated Screening of Natural Inhibitors with protein 7JTY			
**Sr. No.**	**Ligands**	**Compound ID**	**Pyrx Screening Score**
1	Quercetin	5280343	−9.8
2	Quercetin-3-o-glucoside	5280804	−9.6
3	1-caffeoylquinic acid	10155076	−9.2
4	Epicatechin	72276	−8.8
5	Syringic acid	10742	−6.4

**Fig 9:**
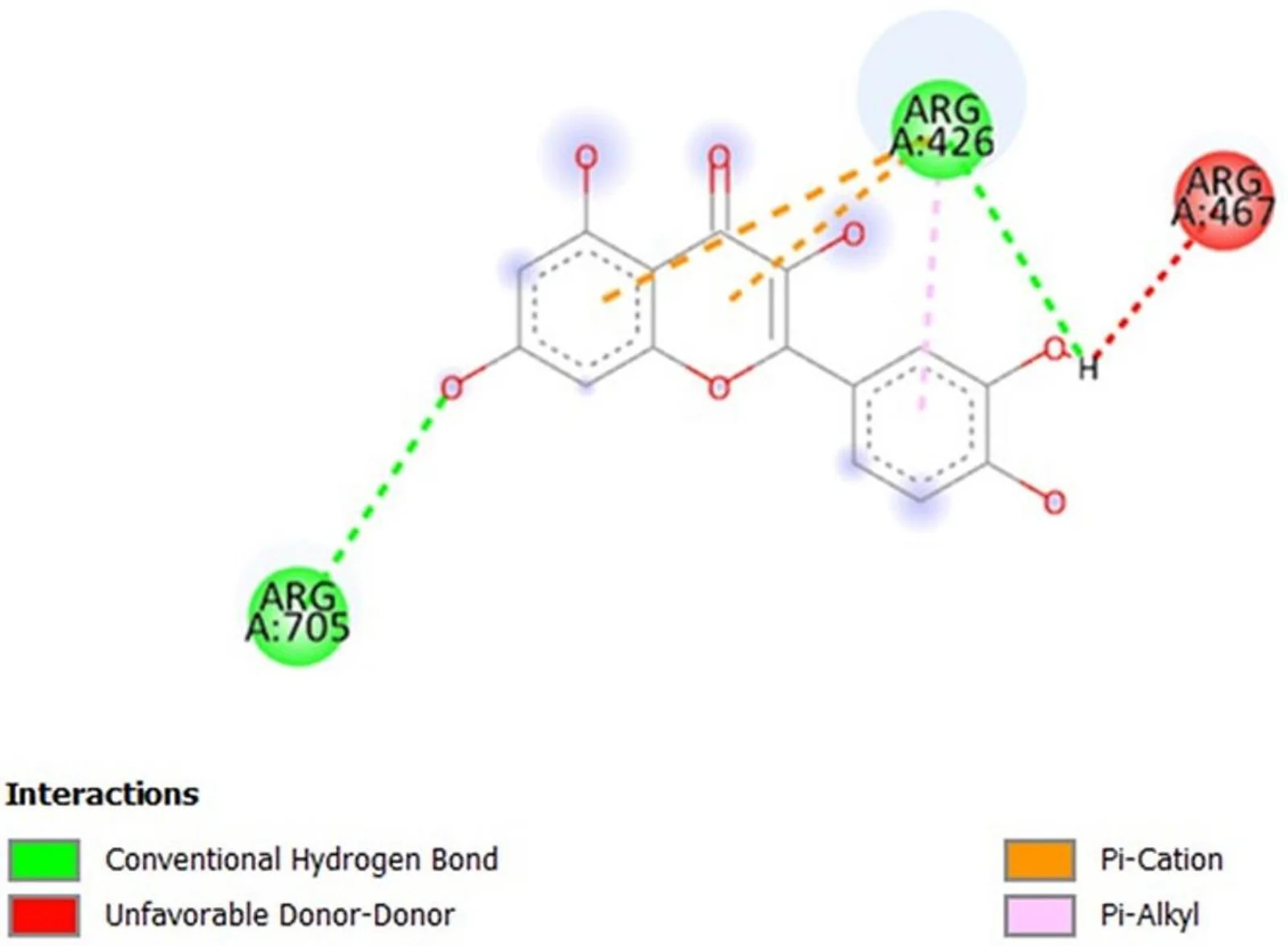
Molecular docking analysis of quercetin with the 7JTY protein. Predicted binding interaction of quercetin with the 7JTY protein generated using PyRx, illustrating the binding pose of the ligand within the active site and its molecular interactions with key amino acid residues.

#### ADMET Profiling of Quercetin.

The pharmacokinetic and safety profile of quercetin was evaluated using the PreADMET online platform, focusing on key ADMET parameters absorption, distribution, metabolism, excretion, and toxicity. This analysis aimed to determine its therapeutic suitability and drug-likeness, following its identification as a potential alpha-glucosidase inhibitor through molecular docking. The drug-likeness of quercetin was assessed using Molinspiration, based on Lipinski’s Rule of Five, a widely accepted guideline for predicting oral bioavailability. The compound demonstrated favorable physicochemical properties, no violations of Lipinski’s criteria. These features suggest good permeability and absorption potential. Additionally, quercetin exhibited promising ADME characteristics, reinforcing its potential as a therapeutic candidate. Comprehensive ADMET data are presented in Table 5.

**Table 5:** SwissADME-predicted physicochemical properties, pharmacokinetic parameters, drug-likeness, and medicinal chemistry profile of quercetin.

ADMET Analysis of quercetin	
**Lipophilicity**	
Log *P*_o/w_ (iLOGP)	1.63
Log *P*_o/w_ (XLOGP3)	1.54
Log *P*_o/w_ (WLOGP)	1.99
Log *P*_o/w_ (MLOGP)	−0.56
Log *P*_o/w_ (SILICOS-IT)	1.54
Consensus Log *P*_o/w_	1.23
**Water Solubility**	
Log S (ESOL)	−3.16
Solubility (ESOL)	2.11e-01 mg/ml; 6.98e-04 mol/l
Log S (Ali)	−3.91
Solubility (Ali)	3.74e-02 mg/ml; 1.24e-04 mol/l
Log S (SILICOS-IT)	−3.24
Solubility (SILICOS-IT)	1.73e-01 mg/ml; 5.73e-04 mol/l
**Pharmacokinetics**	
GI Absorption	High
BBB Permeant	No
P-gp Substrate	No
CYP1A2 Inhibitor	Yes
CYP2C19 Inhibitor	No
CYP2C9 Inhibitor	No
CYP2D6 Inhibitor	Yes
CYP3A4 Inhibitor	Yes
Log *K*p (skin permeation)	−7.05 cm/s
**Druglikeness**	
Lipinski’s Rule	Yes
Ghose’s Rule	Yes
Veber’s Rule	Yes
Egan’s Rule	Yes
Muegge’s Rule	Yes
Bioavailability Score	0.55
**Medicinal Chemistry**	
Pains	Alert: catechol A
Brenk	Alert: catechol
Leadlikeness	Yes
Synthetic Accessibility	3.23

### *In-vivo* analysis

#### Blood glucose level monitoring.

Seventy-two hours following alloxan administration, all treated rats exhibited elevated fasting blood glucose (FBG) levels exceeding 200 mg/dl, confirming successful induction of diabetes, shown in Fig 10. These elevated levels persisted in the untreated diabetic group throughout the study period. In contrast, groups treated with PSE and its nanoparticle formulation (PSENP) showed a progressive reduction in FBG levels. The most notable improvement was observed in the PSENP-treated group at a dose of 70.5 mg/kg body weight, indicating enhanced glycemic control compared to both untreated and standard-treated groups.

**Fig 10:**
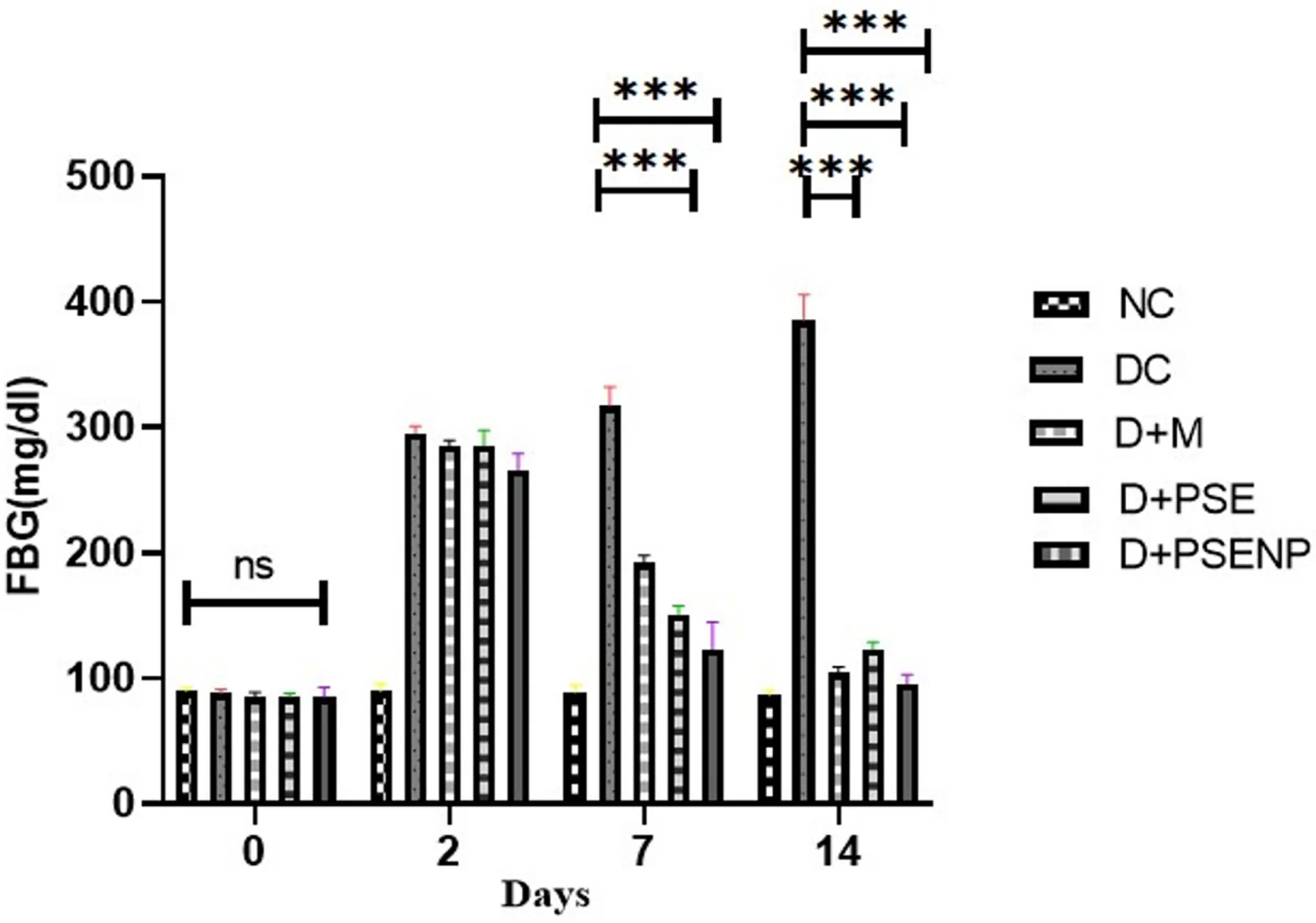
Effect of PSE and PSENP on fasting blood glucose levels in diabetic rats. Fasting blood glucose levels measured in the normal control (NC), diabetic control (DC), diabetic group treated with metformin (D + M), diabetic group treated with PSE (D + PSE), and diabetic group treated with PSENP (D+PSENP) throughout the experimental period. Data are presented as mean ± SD (n = 8). Statistical significance was determined using one-way ANOVA followed by Tukey’s post hoc test. ***p < 0.001 compared with the diabetic control group.

#### Body weight measurement.

A noticeable decline in body weight was observed in rats following alloxan administration, reflecting the typical metabolic disturbances associated with diabetes. However, treatment with PSE and its nanoparticle formulation PSENP led to a gradual improvement in body weight over the study period was shown in Fig 11. This slight but consistent increase suggests a positive effect of the treatments on overall metabolic health and nutrient utilization in diabetic rats.

**Fig 11:**
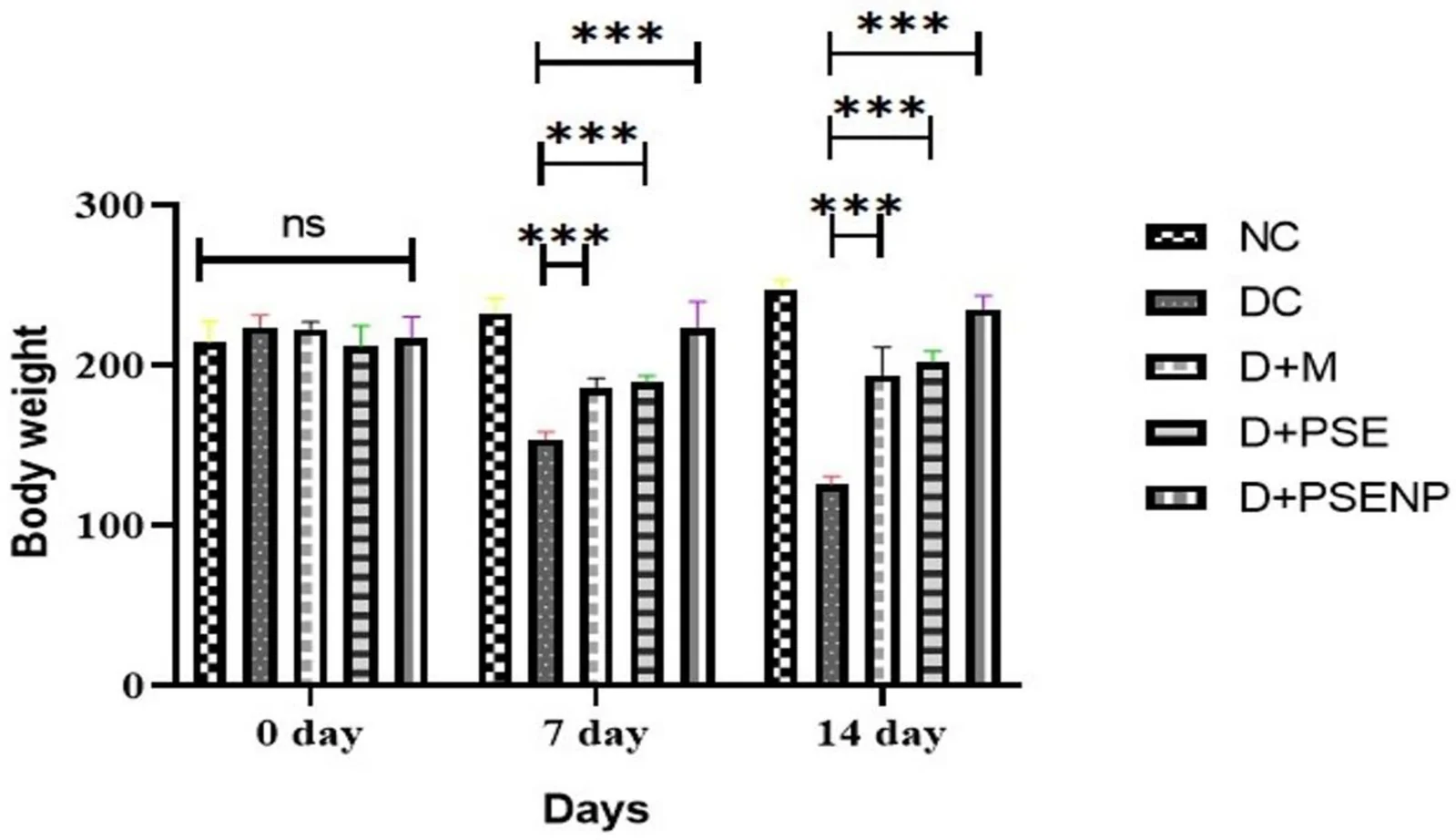
Effect of PSE and PSENP on body weight in diabetic rats. Changes in body weight of the normal control, diabetic control, metformin-treated, PSE-treated, and PSENP-treated groups during the experimental period. Data are presented as mean ± SD (n = 8). Statistical significance was determined using one-way ANOVA followed by Tukey’s post hoc test. ***p < 0.001 compared with the diabetic control group.

#### Serum insulin.

Serum insulin levels were significantly reduced in the untreated diabetic group. However, in Fig 12, administration of PSE and PSENP notably elevated insulin levels compared to the diabetic control, indicating improved pancreatic function.

**Fig 12:**
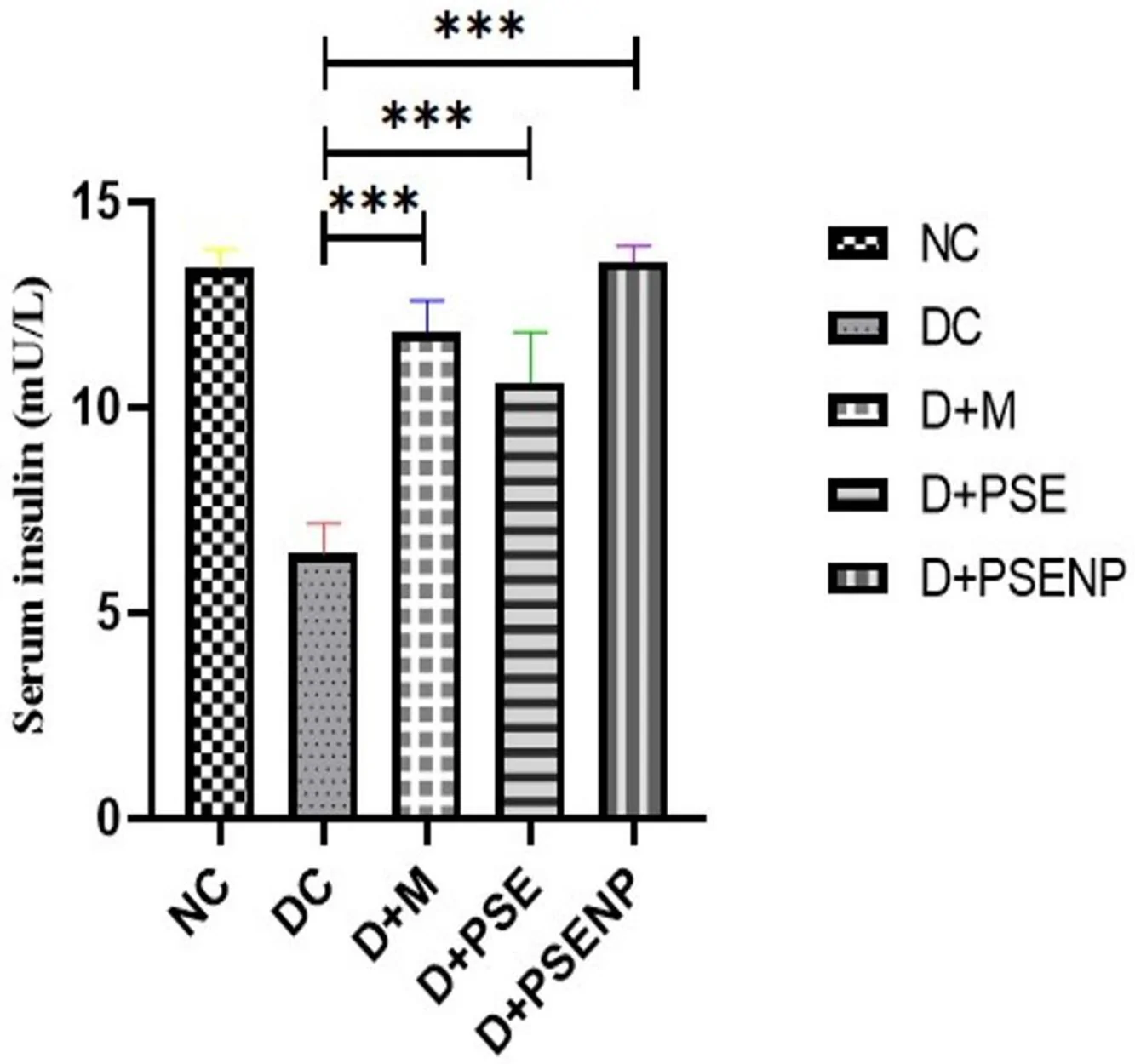
Effect of PSE and PSENP on serum insulin levels in diabetic rats. Serum insulin levels were measured in the normal control, diabetic control, metformin-treated, PSE-treated, and PSENP-treated groups. Data are presented as mean ± SD (n = 8). Statistical analysis was performed using one-way ANOVA followed by Tukey’s post hoc test. ***p < 0.001 compared with the diabetic control group.

### Oxidative stress parameters

Statistically significant differences between the means of the various groups’ total antioxidant capacity (TAC) and total oxidant status (TOS) were found using one-way analysis of variance. In Fig 13, the negative control group’s TAC and TOS results showed that alloxan administration exacerbated oxidative stress. As expected, following treatment with Metformin caused a large increase in TAC, which in turn caused a considerable decrease in TOS levels. TAC levels were observed to be considerably higher with PSE and PSENP than with the negative control group.

**Fig 13:**
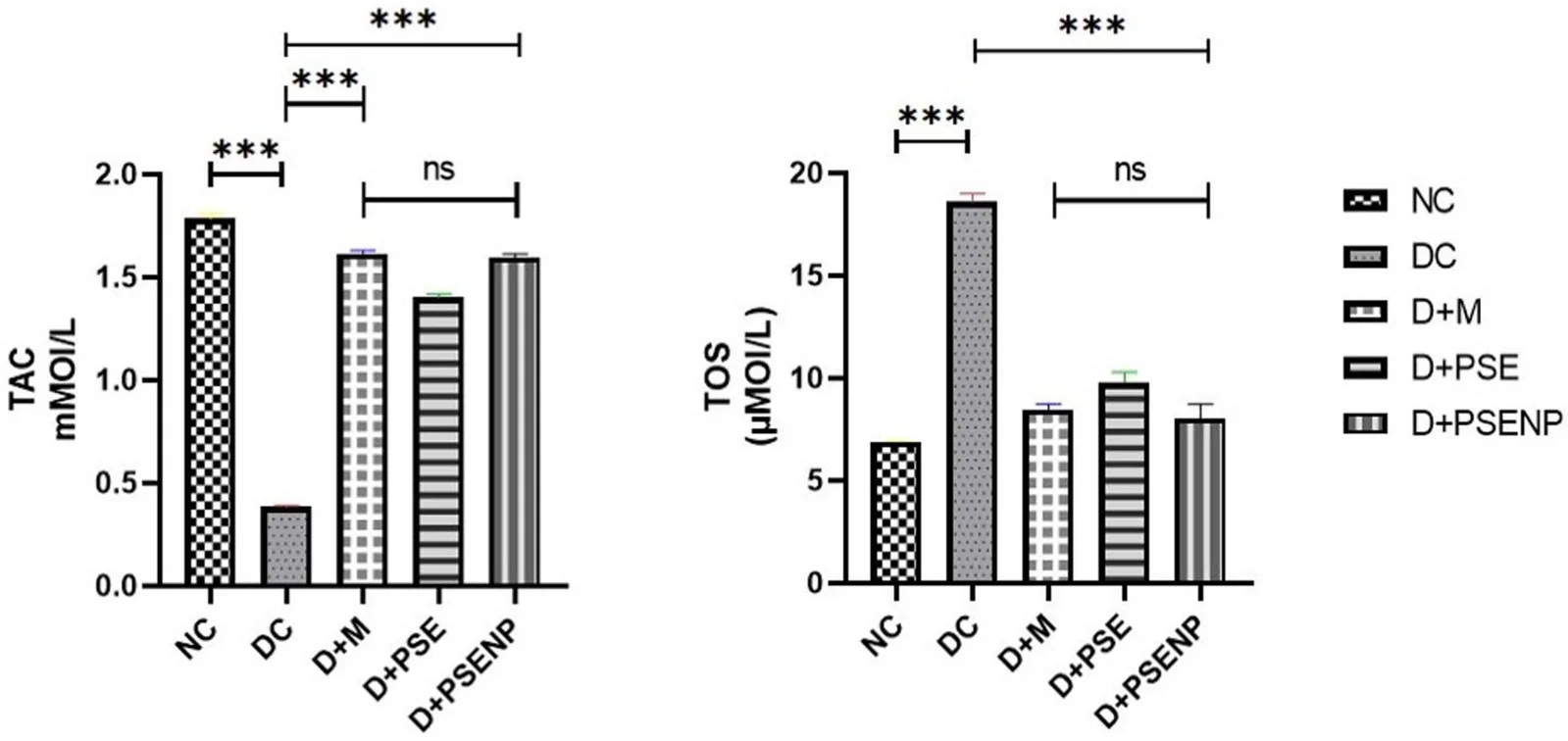
Effect of PSE and PSENP on total antioxidant capacity (TAC) and total oxidant status (TOS) in diabetic rats. (A) Total antioxidant capacity (TAC; mmol Trolox equivalent/L) and (B) total oxidant status (TOS; μmol H_2_O_2_ equivalent/L) measured in the normal control, diabetic control, metformin-treated, PSE-treated, and PSENP-treated groups. Data are presented as mean ± SD (n = 8). Statistical analysis was performed using one-way ANOVA followed by Tukey’s post hoc test. ***p < 0.001 compared with the diabetic control group.

### Anti-oxidant enzyme activity

Assessment of antioxidant enzymes like SOD and CAT was performed to evaluate the oxidative stress status and protective potential of treatments in diabetic conditions. These markers reflect the body’s defense against free radical-induced cellular damage.

The comparative analysis of antioxidant enzymes superoxide dismutase (SOD) and catalase (CAT) is illustrated in Fig 14. Diabetic control rats exhibited a marked decline in both SOD and CAT activity compared to the normal control group, indicating oxidative stress induced by alloxan. However, treatment with metformin, PSE, and particularly PSENP significantly restored SOD and CAT levels. Among the treatments, PSENP-treated rats showed the most notable enhancement in antioxidant enzyme activities, suggesting that nanoparticle encapsulation improved the bioavailability and efficacy of the extract’s antioxidant components

**Fig 14:**
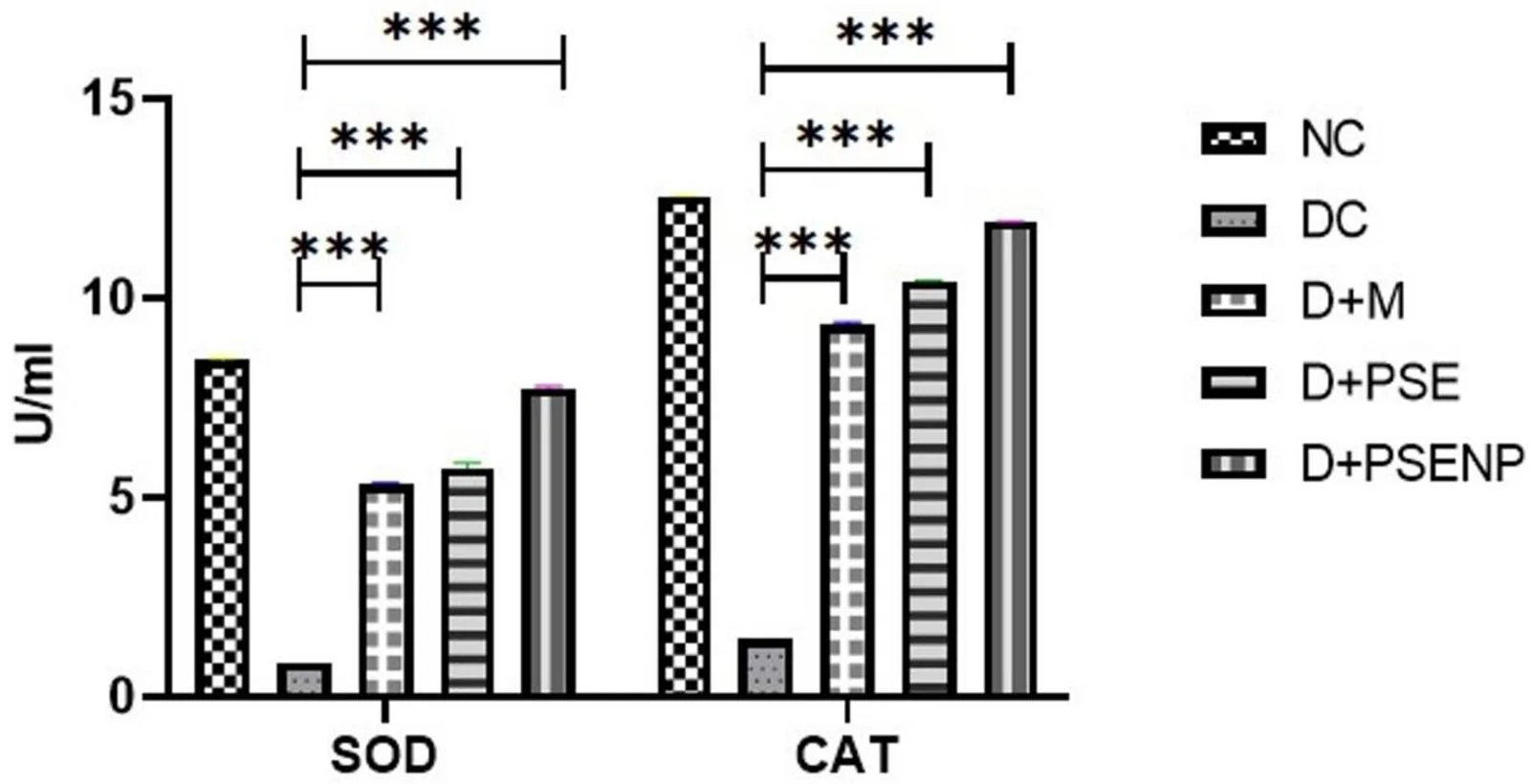
Effect of PSE and PSENP on superoxide dismutase (SOD) and catalase (CAT) activities in diabetic rats. SOD and CAT activities were measured in the normal control, diabetic control, metformin-treated, PSE-treated, and PSENP-treated groups. Data are presented as mean ± SD (n = 8). Statistical analysis was performed using one-way ANOVA followed by Tukey’s post hoc test. ***p < 0.001 compared with the diabetic control group.

### Lipid Profile Modulation by PSE and PSENP Treatment

This analysis was conducted to assess the impact of treatments on lipid metabolism, a critical marker in diabetes-associated dyslipidemia. Diabetic control rats (DC) exhibited significantly elevated levels of total cholesterol (TC), triglycerides (TG), and low-density lipoprotein (LDL), along with markedly reduced high-density lipoprotein (HDL), compared to the normal control (NC) group in Fig 15. Treatment with PSE and PSENP significantly reversed these alterations, lowering TC, TG, and LDL while enhancing HDL levels. PSENP showed a more pronounced improvement than PSE, suggesting better lipid-modulating efficacy, potentially due to improved bioavailability through nanoencapsulation.

**Fig 15:**
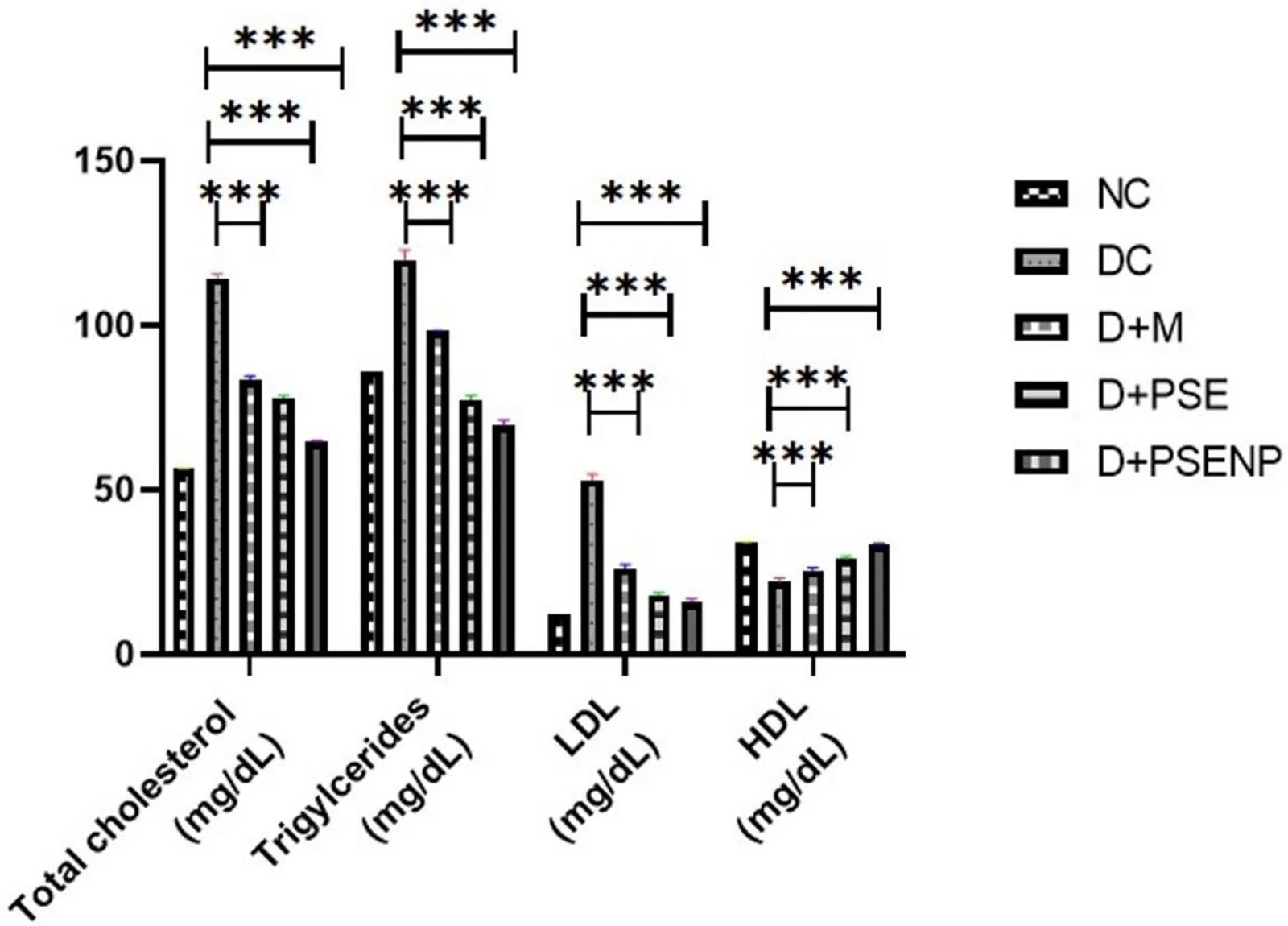
Effect of PSE and PSENP on serum lipid profile in diabetic rats. Serum levels of total cholesterol (TC), triglycerides (TG), low-density lipoprotein cholesterol (LDL), and high-density lipoprotein cholesterol (HDL) were determined in the normal control, diabetic control, metformin-treated, PSE-treated, and PSENP-treated groups. Data are presented as mean ± SD (n = 8). Statistical analysis was performed using one-way ANOVA followed by Tukey’s post hoc test. ***p < 0.001 compared with the diabetic control group.

### Determination of anti-inflammatory markers

The data presented in Fig 16(A) demonstrate a statistically significant increase in the concentrations of inflammatory biomarkers TNF-α and IL-6 in the diabetic groups compared to the normal control groups. Levels of TNF-α and IL-6 reduced in metformin-treated diabetic rats compared to diabetic control rats. Administration of PSE and PSENP to diabetic treated rats led to significantly decreased level of TNF-α and IL-6 when compared to diabetic control. The serum concentration of TNF-α was significantly lower (*P* < 0.001) in the PSE and PSENP-treated rats as compared to the untreated diabetic rats. Additionally, a significantly higher (*P* < 0.001) TNF-α concentration was observed in the untreated diabetic rats when compared to the obese normal control rats.

**Fig 16:**
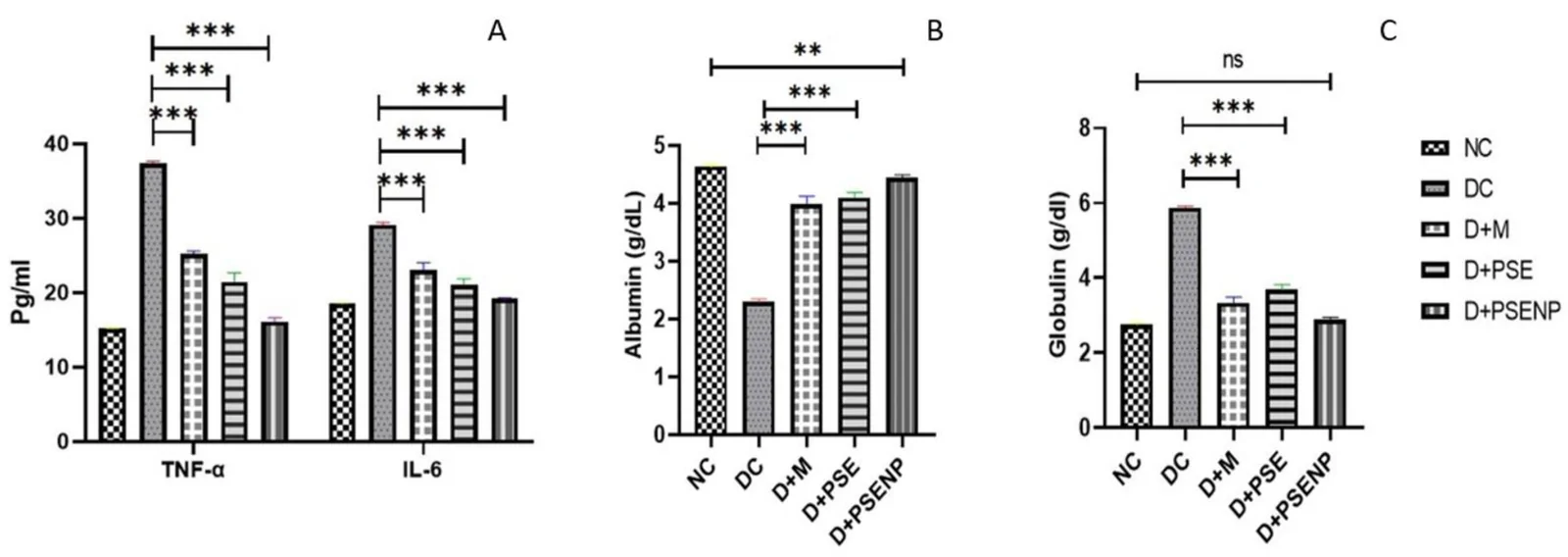
Effect of PSE and PSENP on serum inflammatory cytokines in diabetic rats. (A) Serum concentrations of tumor necrosis factor-alpha (TNF-α) and interleukin-6 (IL-6) in the normal control, diabetic control, metformin-treated, PSE-treated, and PSENP-treated groups. (B) Serum TNF-α levels showing statistically significant differences among the experimental groups. (C) Serum IL-6 levels showing statistically significant differences among the experimental groups. Data are presented as mean ± SD (n = 8). Statistical analysis was performed using one-way ANOVA followed by Tukey’s post hoc test. ***p < 0.001 compared with the diabetic control group.

In Fig 16(B) albumin concentrations significantly reduced (*p* < 0.001) in diabetic rats compared to the normal control. In Fig 16(C), diabetic rats group was significantly decreased the concentration of Globulin. Albumin and globulin levels increased significantly (*p* < 0.001) in PSENP-treated control rats compared to diabetic control rats. In addition, an increase in albumin and globulin level was observed in diabetic treated rats with metformin, PSE and PSENP when compared to diabetic control but with only statistically significant (*p* < 0.001) increase in albumin level in PSENP treated group with compared to diabetic control group.

### Histopathological examinations of pancreas of rats

Following are the histopathological examinations reports of the pancreas from different groups of rat. The Pancreas from various groups were gathered and settled in 10% neutral-buffered formalin (NBF) for the period of 7–10 days, until complete tissue preservation was achieved. Subsequently, the samples were processed for histopathological examination in accordance with the regulations and protocols (Bancroft and Gamble, 2007). All the slide findings were analysed and described fully and interpreted appropriately as shown in Fig 17.

**Fig 17:**
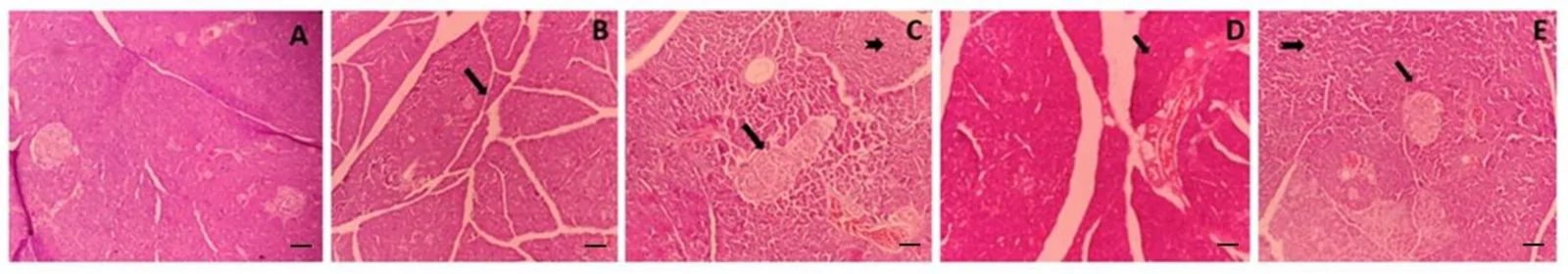
Histopathological examination of pancreatic tissues stained with hematoxylin and eosin (H&E) and examined under a light microscope at 10 × magnification. A) The pancreatic tissue of the normal control group showed normal architecture of pancreatic histology. The pancreas demonstrated usual islets of Langerhans, characterized by centrally located, pale, round to ovoid β-cells (arrow) situated within the exocrine tissue of the pancreas. Acinar cells were present in normal architecture with centrally placed nuclei. The pancreatic duct also exhibited normal histology, with the lumen lined by a single layer of cuboidal ductal epithelial cells. The collagenous duct wall was well preserved, and the lumen appeared collapsed and empty, consistent with normal pancreatic histological features. B) The pancreatic tissue of diabetic rats showed marked narrowing and degeneration of the islets of Langerhans with necrosis of the component cells. The nuclei appeared densely basophilic and karyolytic. Acinar cells also exhibited necrotic changes accompanied by cellular swelling. At several locations, the pancreatic ducts showed necrosis and distortion of the ductal epithelium. In addition, vascular congestion was evident. Overall, severe disruption of normal pancreatic architecture was observed in the diabetic control group. C) The pancreatic section of the metformin-treated group showed usual islets of Langerhans characterized by pale, large, round to ovoid-shaped cells (arrow) situated within the exocrine tissue of the pancreas. Acinar cells were in good condition and exhibited normal histological structure, closely resembling those of the normal control group. The pancreatic ducts maintained normal morphology and epithelial integrity. Overall, all pancreatic structures exhibited near-normal architecture, indicating statistically significant restoration of pancreatic tissue following metformin treatment. D) Histopathological investigation of the pancreatic tissue from the PSE treated group revealed usual islets of Langerhans; however, shrunken islets were observed at certain locations. Mild necrosis of cells was also present. Acinar cells generally maintained normal morphology, although some cells showed swelling. The pancreatic duct exhibited focal necrotic changes. Despite these alterations, the overall histological appearance demonstrated considerable improvement compared with the diabetic control group. E) The histopathological section of pancreatic tissue from the PSENP treated group showed usual islets of Langerhans composed of round to ovoid cells which are large, pale and situated within the exocrine tissue of the pancreas. Acinar cells showed normal histological and their structur is also found comparable with the normal control group. The pancreatic ducts retained normal epithelial integrity and architecture. Overall, the pancreatic tissue showed marked restoration of normal histological features with minimal pathological alterations.

### Evaluation of safety profile of PSENP

#### Histopathological evaluation of the safety profile of PSENP.

The safety profile of the prepared PSENP was evaluated through histopathological examination of liver and kidney tissues following administration for 1, 3, and 7 days.

Histological analysis of liver sections revealed no statistically significant treatment-related pathological alterations throughout the study period (Fig 18A–C). On day 1, normal hepatic architecture was observed, characterized by well-organized hepatic cords and normal arrangement of hepatocytes around the central vein (Fig 18A). Similarly, liver sections examined on day 3 demonstrated normal hepatocytes with centrally located nuclei, regular sinusoidal spaces, and absence of inflammatory cell infiltration (Fig 18B). On day 7, hepatic tissue retained normal morphology with healthy hepatocytes and centrally placed nuclei; however, occasional pyknotic nuclei were observed without evidence of tissue degeneration or inflammatory changes (Fig 18C).

**Fig 18:**
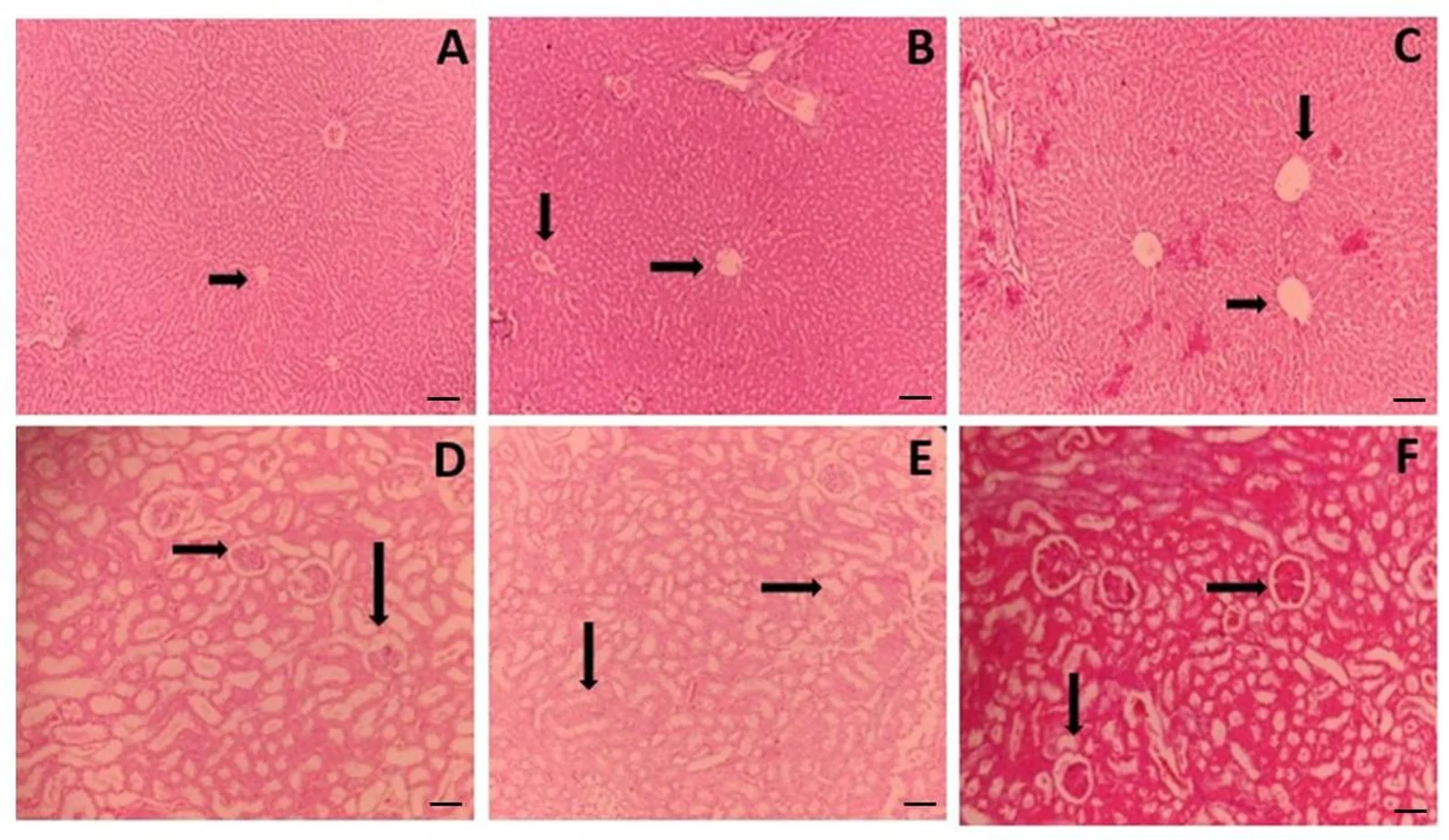
Histopathological evaluation of the safety profile of PSENP in liver and kidney tissues. Representative hematoxylin and eosin (H&E)-stained sections examined under a light microscope (10 × objective magnification). (A–C) Liver sections collected on days 1, 3, and 7, respectively, showing preserved hepatic architecture with normal hepatocytes, hepatic cords, and sinusoidal spaces without evidence of statistically significant pathological alterations. (D–F) Kidney sections collected on days 1, 3, and 7, respectively, showing normal renal histology with intact glomeruli and renal tubules, preserved tissue organization, and absence of notable degenerative changes. Arrows indicate representative hepatic and renal structures. These findings suggest that PSENP administration did not induce observable hepatic or renal toxicity during the study period.

Histopathological examination of kidney sections also indicated the absence of statistically significant nephrotoxic effects following PSENP administration (Fig 18D–F). On day 1, normal renal architecture was observed, including intact glomeruli and renal tubules with normal glomerular thickness and symmetry (Fig 18D). A few scattered lymphocytic cells were noted without associated tissue damage. Kidney sections from day 3 showed normal glomeruli, blood vessels, renal tubules, and interstitial tissue. The proximal convoluted tubules, distal tubules, and loop of Henle exhibited preserved structural organization and normal morphology (Fig 18E). By day 7, renal tissues maintained normal histological features with intact glomeruli and tubules, normal glomerular thickness, and no evidence of tubular degeneration or necrosis (Fig 18F).

Overall, the histopathological findings demonstrated that PSENP administration did not induce any notable hepatic or renal toxicity, supporting the biocompatibility and safety of the developed nanoparticle formulation.

## Discussion

The present study demonstrates that PLGA-based nanoencapsulation of *Persea americana* seed extract significantly enhances its antihyperglycemic, antioxidant, anti-inflammatory, and antihyperlipidemic activities in an alloxan-induced diabetic rat model. The superior therapeutic effects observed with PSENP may be associated with modulation of oxidative stress and inflammatory responses, as evidenced by the statistically significant improvement in antioxidant enzyme activities and reduction in pro-inflammatory cytokines. HPLC analysis identified several bioactive phytochemicals, including quercetin, epicatechin, quercetin-3-O-glucoside, syringic acid, and caffeoylquinic acid derivatives, which have previously been reported to possess antioxidant and antidiabetic properties.

Dynamic light scattering confirmed successful incorporation of PSE into the PLGA matrix, as evidenced by an increase in particle size from 312.6 nm (blank PLGA) to 471.2 nm (PSENP). Although this particle size is larger than that typically preferred for parenteral nanocarriers, it remains suitable for oral delivery systems in previously reported studies [38–40]. In the gastrointestinal environment, submicron PLGA-based systems can prolong residence time, protect bioactive compounds from enzymatic and oxidative degradation, and enable sustained release, thereby maintaining prolonged exposure of phytoconstituents at the absorption site [18,41] and hence contributing superior *In vivo* efficacy observed with PSENP compared with crude extract.

The improved physicochemical stability of PSENP was further supported by a lower polydispersity index and increased negative zeta potential, indicating a more uniform and stable colloidal system. The presence of phenolic and acidic groups within the extract likely contributed to enhanced electrostatic stabilization, reducing aggregation and improving interaction with the intestinal mucosa. These properties may facilitate improved epithelial uptake and transport of bioactive compounds through endocytic pathways [19,42].

The FTIR results suggest that hydrogen bonding between hydroxyl-rich phytoconstituents and the carbonyl groups of PLGA facilitated stable encapsulation while preserving polymer integrity, consistent with the molecular interactions reported for PLGA-based phytochemical delivery systems [43].

The spherical morphology observed via TEM for both PLGANP and PSENP is consistent with previously reported PLGA-based nanoparticle systems, where emulsion–solvent evaporation techniques typically yield smooth, uniformly shaped particles. Similar studies have also documented an increase in particle size following incorporation of plant-derived extracts into PLGA matrices, which has been attributed to successful encapsulation of bioactive constituents within the polymeric core. Slight aggregation observed in TEM images has likewise been reported in the literature and is commonly associated with sample drying during grid preparation rather than true instability of the colloidal system [44]. Moreover, the stable turbidity profile indicates minimal nanoparticle aggregation, reflecting the inherent colloidal stability of the PLGA matrix, consistent with previous reports on PLGA-based nanosystems [45]. Overall, the present findings are in agreement with earlier reports, supporting the reliability of the formulation method used in this study.

Phytochemical profiling confirmed the presence of quercetin, epicatechin, quercetin-3-O-glucoside, syringic acid, and caffeoylquinic acid derivatives, all of which possess well-documented antioxidant and antidiabetic properties [46]. Among these, quercetin demonstrated strong binding affinity toward α-glucosidase in molecular docking analysis, suggesting a potential mechanism for delayed carbohydrate digestion and reduced postprandial glucose absorption [20,47,48].

Nanoencapsulation modestly increased the total phenolic and flavonoid content detected in PSENP, suggesting protection of phytoconstituents from oxidative degradation. The polymer matrix likely preserved redox-sensitive functional groups and improved their stability, enhancing biological accessibility. Although the increase was relatively small, improved phytochemical preservation may have contributed to the enhanced biological activity observed in PSENP [15,49]. Thus, the enhanced retention of phenolic and flavonoid compounds following PLGA encapsulation suggests improved protection of phytoconstituents against degradation, consistent with previous reports on PLGA-based delivery systems [50].

The *in silico* findings further support the antidiabetic potential of the identified phytoconstituents. Quercetin exhibited the highest binding affinity toward α-glucosidase (PDB ID: 7JTY), indicating favorable interactions with the enzyme’s active site. These findings agree with previous reports demonstrating strong α-glucosidase inhibition by quercetin and its derivatives [51,52]. Furthermore, the favourable ADMET profile suggests desirable drug-like properties. Although computational analyses are predictive, they complement the observed biological activity of PSENP and support its therapeutic potential.

*In vivo*, PSENP demonstrated superior glycemic control compared with crude extract. Alloxan induces diabetes primarily through reactive oxygen species-mediated destruction of pancreatic β-cells, leading to insulin deficiency and sustained hyperglycemia [4,5,53]. The enhanced efficacy of PSENP may be attributed to sustained release of antioxidant phytochemicals, which continuously mitigate oxidative stress, preserve residual β-cell function, and support insulin secretion [54].

Restoration of serum insulin levels and body weight further indicates improved metabolic control. Polyphenols such as quercetin and epicatechin can attenuate oxidative stress and inhibit apoptotic signaling pathways, while nanoparticle delivery enhances absorption and systemic availability [9,55].

Oxidative stress contributes to inflammation via activation of NF-κB signaling. In diabetic controls, elevated ROS was associated with impaired antioxidant enzymes. PSENP significantly increased superoxide dismutase and catalase activity, indicating improved enzymatic defense and radical scavenging capacity [56].

PSENP also reduced TNF-α and IL-6 levels, likely through inhibition of NF-κB-mediated cytokine expression [2,3]. Enhanced delivery and sustained release of polyphenols may strengthen this anti-inflammatory effect.

Improvement in lipid profiles may be linked to enhanced insulin activity, reduced oxidative modification of lipoproteins, and modulation of hepatic lipid metabolism. Sustained phytochemical exposure likely contributed to lipid normalization.

Histopathological evaluation confirmed biochemical findings, demonstrating preservation of pancreatic islet architecture in PSENP-treated animals. This protective effect is likely mediated through attenuation of oxidative stress and inflammatory damage, thereby reducing β-cell degeneration and supporting functional recovery. The absence of histological toxicity in liver and kidney tissues further supports the biocompatibility and safety of the developed nanoformulation.

Collectively, these findings indicate that the therapeutic superiority of PSENP is driven not only by enhanced phytochemical stability but also by its ability to show biological activity,strengthening its translational potential in diabetes management.

### Limitations of the study

This study has certain limitations that should be acknowledged. First, the absence of a comparative pharmacokinetic evaluation between crude extract and PSENP limits direct conclusions regarding improved systemic bioavailability. Second, the use of an alloxan-induced diabetic model primarily reflects a Type 1-like diabetic condition characterized by β-cell destruction; therefore, the findings may not fully represent insulin-resistant Type 2 diabetes [4,5]. Third, although molecular docking and *In vitro* assays suggest possible mechanisms, experimental validation of signaling pathways such as NF-κB was not performed. Finally, particle size optimization and long-term biodistribution studies were beyond the scope of the present work but are warranted in future investigations.

Although PSENP exhibited a relatively large particle size (~471 nm) compared with conventional parenteral nanocarriers, the formulation was designed for oral administration and demonstrated statistically significant therapeutic efficacy. These findings suggest that the stability and physicochemical characteristics of nanoformulation are key determinants of its *in vivo* performance rather than rapid systemic distribution. Collectively, the findings indicate that the developed nanoformulation represents a robust nanocarrier system capable of achieving enhanced therapeutic efficacy.

## Conclusion

The present study demonstrated that PLGA-based nanoencapsulation enhanced the therapeutic efficacy of *Persea americana* seed extract in an alloxan-induced diabetic rat model. PSENP showed superior antihyperglycemic, antioxidant, anti-inflammatory, and antihyperlipidemic effects compared with the crude extract, accompanied by improved insulin levels, restoration of antioxidant defenses, reduced inflammatory cytokines, and preservation of pancreatic architecture.

The enhanced biological activity of PSENP may be attributed to improved phytochemical stability and sustained release provided by the PLGA carrier system. However, given the use of an alloxan-induced type 1-like diabetic model and the absence of pharmacokinetic evaluation, further studies are needed to investigate its bioavailability, efficacy and molecular mechanism in other diabetic models. Overall, PSENP represents a promising nanoformulation for improving the therapeutic potential of *P. americana* seed extract in experimental diabetes.

## Supporting information

S1 File Supporting data underlying the physicochemical characterization, drug-release profile, in vitro bioactivity assays, and in vivo biochemical evaluation of PLGA nanoparticles encapsulating *Persea americana* seed extract. (DOCX)
