# Supplementary material for: Polylactic co-glycolic acid (PLGA)-based nanoparticles encapsulating Persea americana seed extract: A potential therapeutic strategy against oxidative stress and diabetes in rat model
Source: PLoS One. 2026 Oct 22;21(10):e0359514. doi: 10.1371/journal.pone.0359514 (PMC13690202; doi:10.1371/journal.pone.0359514)
Supplement: Supplementary file 1 [file pone.0359514.s001.docx]

**Encapsulation Efficiency and Drug Loading**

| Time (mins) | % drug released | | |
| --- | --- | --- | --- |
| 0 | 0 | 0 | 0 |
| 2 | 18.4 | 18.4 | 18.5 |
| 4 | 32.7 | 32.7 | 32.8 |
| 6 | 45.2 | 45.2 | 45.2 |
| 8 | 56.8 | 56.8 | 56.8 |
| 12 | 68.3 | 68.3 | 68.3 |
| 24 | 79.6 | 79.6 | 79.6 |
| 48 | 91.2 | 91.2 | 91.2 |

**Total phenolic content and total flavonoid content determination**

|  | PSE | | | PSENP | | | Quercetin/Gallic acid | | |
| --- | --- | --- | --- | --- | --- | --- | --- | --- | --- |
| TPC (mg GAE/g) | 252.9 | 252.99 | 253.09 | 268.87 | 268.96 | 269.05 | 285.9 | 285.99 | 285.09 |
| TFC (mg QUE/g) | 245.92 | 245.97 | 246.15 | 255.6 | 255.85 | 255.93 | 282.9 | 282.99 | 283.09 |

**Alpha-Glucosidase Inhibition and DPPH Radical Scavenging Activity**

|  | PSE | | | PSENP | | | Acarbose | | |
| --- | --- | --- | --- | --- | --- | --- | --- | --- | --- |
| alpha-gylcosidase | 58.41 | 58.43 | 58.45 | 72.56 | 72.57 | 72.59 | 76.32 | 76.33 | 76.34 |
| DPPH | 36.64 | 36.68 | 37.02 | 48.45 | 48.47 | 48.49 | 76.32 | 76.33 | 76.34 |

**Blood Glucose Level Monitoring**

**Body Weight Measurement**

**Serum Insulin**

| NC |  | DC |  | D+M |  | D+PSE |  | D+PSENP |  |
| --- | --- | --- | --- | --- | --- | --- | --- | --- | --- |
| 13.40 ± 0.46 | 8 | 6.46 ± 0.74 | 8 | 11.83 ± 0.67 | 8 | 10.61 ± 1.40 | 8 | 13.55 ± 0.40 | 8 |

**Oxidative stress parameters**

**TAC**

**TOS**

| NC | DC | D+M | D+PSE | D+PSENP |
| --- | --- | --- | --- | --- |
| 6.95 | 18.809 | 9.021 | 10.23 | 8.54 |
| 6.9 | 17.95 | 8.35 | 9.27 | 8.002 |
| 6.86 | 18.818 | 8.27 | 10.04 | 7.111 |
| 6.851 | 18.822 | 8.21 | 10.128 | 8.435 |
| 6.907 | 18.806 | 8.496 | 9.14 | 7.297 |
| 6.882 | 18.817 | 8.463 | 10.218 | 8.333 |
| 6.869 | 17.842 | 8.478 | 10.126 | 7.345 |
| 6.876 | 18.819 | 8.488 | 9.172 | 9.112 |

**Anti-oxidant enzyme activity**

**Lipid Profile Modulation by PSE and PSENP Treatment**

**Determination of anti-inflammatory markers**

|  | **NC** |  |  | **DC** |  |  | **D+M** |  |  | **D+PSE** |  |  | **D+PSENP** |  |  |
| --- | --- | --- | --- | --- | --- | --- | --- | --- | --- | --- | --- | --- | --- | --- | --- |
| TNF-a | 15.1951 | 0.05610 | 8 | 37.4063 | 0.26136 | 8 | 25.25 | 0.32513 | 8 | 21.467 | 1.20362 | 8 | 16.15 | 0.46904 | 8 |
| IL-6 | 18.5523 | 0.05598 | 8 | 29.1287 | 0.29873 | 8 | 23.035 | 1.02025 | 8 | 21.072 | 0.80965 | 8 | 19.1762 | 0.18897 | 8 |

**Albumin level**

| NC | DC | D+M | D+PSE | D+PSENP |
| --- | --- | --- | --- | --- |
| 4.62 | 2.34 | 3.98 | 4.1 | 4.43 |
| 4.62 | 2.35 | 4.2 | 4.23 | 4.46 |
| 4.63 | 2.33 | 3.84 | 4.18 | 4.36 |
| 4.57 | 2.23 | 3.87 | 3.99 | 4.48 |
| 4.68 | 2.25 | 4.09 | 4 | 4.42 |
| 4.56 | 2.32 | 4.12 | 4.11 | 4.39 |
| 4.64 | 2.3 | 3.91 | 4.01 | 4.51 |
| 4.69 | 2.26 | 3.83 | 4.16 | 4.47 |

**Globulin level**

| **NC** | **DC** | **D+M** | **D+PSE** | **D+PSENP** |
| --- | --- | --- | --- | --- |
| 2.74 | 5.87 | 3.33 | 3.79 | 2.86 |
| 2.75 | 5.88 | 3.44 | 3.54 | 2.79 |
| 2.72 | 5.9 | 3.03 | 3.84 | 2.94 |
| 2.74 | 5.85 | 3.23 | 3.63 | 2.86 |
| 2.73 | 5.86 | 3.28 | 3.84 | 2.94 |
| 2.76 | 5.88 | 3.52 | 3.46 | 2.88 |
| 2.86 | 5.84 | 3.39 | 3.73 | 2.87 |
| 2.72 | 5.94 | 3.45 | 3.57 | 2.98 |
